# Lower early mortality and risk prediction improvement of obesity after acute pulmonary embolism: results from a multicenter cohort analysis with external validation

**DOI:** 10.1016/j.rpth.2025.102718

**Published:** 2025-02-28

**Authors:** Romain Chopard, Laurent Bertoletti, Marc Badoz, Nicolas Meneveau, Fiona Ecarnot, Luciano López Jiménez, Olga Madridano, José Antonio Díaz Peromingo, Meritxell López De la Fuente, Manuel Monreal, Gregory Piazza, RIETE Investigators

**Affiliations:** 1 Department of Cardiology, University Hospital Besançon, Besançon, France; 2 SINERGIES Laboratory, University Marie & Louis Pasteur, Besançon, France; 3 F-CRIN, INNOVTE network, France; 4 Université Jean Monnet Saint-Étienne, CHU Saint-Étienne, Mines Saint-Etienne, INSERM, SAINBIOSE U1059, CIC 1408, Département of Médecine Vasculaire et Thérapeutique, Saint-Etienne, France; 5 Internal Medicine Department, Hospital Universitario Reina Sofía, Córdoba, Spain; 6 Department of Internal Medicine, Hospital Infanta Sofía, Madrid, Spain; 7 Department of Internal Medicine, Hospital Clínico Universitario de Santiago, Santiago de Compostela, Spain; 8 Hospital Universitari Mútua de Terrassa, Terrassa, Cataluña, Spain; 9 Cátedra de Enfermedad Tromboembólica, Universidad Católica de Murcia, Murcia, Spain; 10 CIBER de Enfermedades Respiratorias (CIBERES), Instituto de Salud Carlos III, Madrid, Spain; 11 Division of Cardiovascular Medicine, Department of Medicine, Brigham and Women’s Hospital, Harvard Medical School, Boston, Massachusetts, USA

**Keywords:** obesity, outcomes, pulmonary embolism

## Abstract

**Background:**

The relationship between obesity (defined as body mass index [BMI] ≥ 30 kg/m^2^) and mortality in venous thromboembolism remains controversial.

**Objectives:**

We aimed to compare outcomes after pulmonary embolism (PE) between patients with obesity and nonobese, nonunderweight patients.

**Methods:**

Using a multicenter registry of prospectively recorded individual patient data, we compared outcome rates using multivariable logistic or Cox regression for 30-day and 6-month outcomes respectively (etiologic analysis). We assessed the incremental value of adding BMI information on top of the 30-day European Society of Cardiology (ESC) prognostic algorithm (prognostic analysis).

**Results:**

We included 2390 patients with BMI of ≥18.5 kg/m^2^ (mean age, 66.9 ± 16.8 years; 1188 men [49.7%]); 686 patients [28.7%] were in the obese group. Mortality rates were significantly lower in patients with obesity than that in patients who were nonobese at 30 days (3.2% [95% CI, 2.0-4.8] vs 5.9% [95% CI, 4.8-7.1]), and 6 months (8.1% [95% CI, 6.2-10.4] vs 16.3% [95% CI, 14.6-18.1]). Rates of secondary nonfatal outcomes (including bleeding, recurrent venous thromboembolism, myocardial infarction, and stroke) did not differ between groups. The addition of the obesity information on top of the ESC prognostic model improved global model fit and discriminatory (Harrell C index from 0.636 to 0.657; *P* = .07) and calibration capacities (*P* (Hosmer–Lemeshow) = .02 vs .13), yielding significant reclassification (ie, 10.3%) based on the observed mortality rates with the ESC model as reference. Findings were confirmed in an external validation using 35,796 patients with PE from the RIETE registry.

**Conclusion:**

We present evidence indicating lower early- and mid-term mortality after PE in patients classified as obese based on BMI, compared with nonobese, nonunderweight patients. BMI should likely be incorporated into algorithms or scoring systems for predicting early mortality following PE.

## Essentials

• The link between obesity and mortality in pulmonary embolism (PE) remains a debated topic.
• We analyzed 2390 patients with PE from a regional registry, validated in RIETE.
• The 30-day and 6-month mortality rates were lower in obese patients, with similar rates of other outcomes.
• The addition of the obesity information improved risk prediction for early mortality.
• Obesity should likely be included in algorithms to predict early mortality after PE.

## 1 Introduction

Over the last 3 decades, the worldwide prevalence of obesity has nearly doubled, and mean body mass index (BMI) has increased worldwide by 0.4 kg/m^2^ per decade for men and 0.5 kg/m^2^ per decade for women [1]. Obesity is associated with mortality with more than 500,000 deaths per year in the United States and a loss in life expectancy of nearly 2.4 years, in the overall population [2].

Population-based studies have shown that obesity is a minor persistent risk factor for venous thromboembolism (VTE), with patients with obesity having a 2-fold higher risk of developing incidental and recurrent deep vein thrombosis and/or pulmonary embolism (PE) compared with patients who were nonobese [[3], [4], [5]]. Obese patients have an increased risk of hospitalization (relative risk, 1.39; 95% CI, 1.25-1.55) compared with nonobese patients [6]. The association of obesity with VTE has been strengthened by Mendelian randomization studies of genetic variants associated with increased adiposity [7,8]. In addition to the VTE risk associated with higher BMI, childhood obesity was associated with increased risk in adulthood, independent of BMI in adulthood [9]. Given the increased risk of VTE as well as cardiometabolic risk observed in patients with obesity, a higher mortality is typically expected. However, a counterintuitive paradoxical survival benefit of obesity has been observed in some VTE studies, whereby mortality from PE has been found to be decreased in patients with obesity compared with that in lean counterparts [[10], [11], [12], [13], [14], [15]]. The relationship between obesity and mortality in VTE thus remains controversial, and the mechanisms mediating the effect are still not well understood [16,17].

We used data from a prospective multicenter cohort: (1) to compare early outcomes (at 30 days and 6 months) after acute PE between patients with obesity (BMI ≥ 30 kg/m^2^) and those with normal-to-overweight BMI and (2) to assess the incremental prognostic value of adding BMI information on top of the European Society of Cardiology (ESC) algorithm for the prediction of 30-day mortality [18]. We performed an external validation of the primary outcome and additional risk prediction analyses in the Computerized Registry of Patients with Venous Thromboembolism (RIETE) population.

## 2 Methods

### 2.1 Study design

We performed a post hoc analysis based on prospectively recorded individual patient data from the Burgundy Franche-Comté FRANCE registry. Patients were included between January 2011 and July 2021. Briefly, the Burgundy Franche-Comté FRANCE registry is an ongoing, noninterventional, multicenter registry (6 French centers including 2 tertiary care facilities and 4 general, nonacademic hospitals) that prospectively records baseline characteristics and follow-up of consecutive patients with a confirmed diagnosis of acute PE [19]. The registry received approval from the national commission for data privacy and protection. This study was conducted in accordance with the amended Declaration of Helsinki. Our institutional review board approved the study protocol. All patients provided written informed consent for participation in the registry in accordance with local ethics committee requirements. If the patient was unable to give consent, especially in case of cardiac arrest, the family (or legal guardian) was asked for authorization to collect and use clinical and outcomes data.

### 2.2 Patient selection

Inclusion criteria were patients aged 18 years or older with a diagnosis of both symptomatic and incidental PE confirmed by computed tomography pulmonary angiography or ventilation-perfusion scan [20,21]. There were no exclusion criteria. Management was at the discretion of the physician in charge and was in accordance with the guidelines in place at the time of the study [18,[22], [23], [24]].

### 2.3 Data collection

Research physicians (not involved in the care of the patients included) prospectively entered data into a dedicated database for deidentification of personal information and for data validation. Research physicians were also asked to ensure that recruitment was consecutive. Patients returned to the participating centers at 6 months after the initial phase for a follow-up visit to record clinical status, anticoagulation treatments, and outcomes. If the patients did not attend the hospital visit, physicians followed the sequential procedure hereafter: conducted telephone interview with the patient (or family), consulted the hospitalization records, contacted the patient’s general practitioner, and consulted the national death registry.

### 2.4 Study end points and definitions

Primary outcomes were all-cause mortality at 30 days and 6 months. Secondary outcomes included the following: major bleeding and clinically relevant nonmajor (CRNM) bleeding at 30 days and major bleeding, CRNM bleeding, recurrent VTE, acute myocardial infarction (MI), and acute stroke at 6 months. All suspected outcome events were classified by a central adjudication committee comprising 2 cardiovascular medicine specialists (R.C., N.M.), blinded to patient status (obesity or no obesity). The criteria for the diagnosis and definitions for adjudication of all outcomes and their components are described in the Supplementary Material.

Obesity was categorized according to the World Health Organization (WHO) definition as BMI of ≥30 kg/m^2^ [25]. Patients with obesity were compared with nonobese, nonunderweight patients, including both those with normal weight (ie, BMI, 18.5-24.9 kg/m^2^) and those who were overweight (ie, BMI, 25.0-29.9 kg/m^2^) according to the WHO definition [25]. We excluded from the analysis underweight patients (BMI < 18.5 k/m^2^) whose body weight was considered too low to be healthy (eg, malignancy, sarcopenia, chronic illness, chronic infection, inflammation, substance abuse, and malnutrition) and therefore would not be a fair or informative comparator for patients with obesity [25]. Indeed, underweight patients are very likely to be phenotypically different [13], and their exclusion removes confounding in the reference group, in line with the precedent set in a previous study [13].

PE was risk-stratified, and right ventricular dysfunction was defined according to the ESC 2019 guidelines (Supplementary Material) [18]. Positive troponin was defined as a value >99th percentile of healthy subjects with a coefficient of variation of 10% [26]. Components of the simplified Pulmonary Embolism Severity Index were recorded for all patients and are summarized in Supplementary Table S1 [27].

### 2.5 Statistical analysis

Based on the Recommendations for Statistical Reporting in Cardiovascular Medicine from the American Heart Association [28], we report the study methods and results for the etiologic analyses in accordance with the Strengthening the Reporting of Observational studies in Epidemiology (STROBE) guidelines. The prognostic analysis followed guidelines from the transparent reporting of a multivariable prediction model for individual prognosis or diagnosis statement [29]. Continuous variables are reported as mean ± SD or median and IQR, as appropriate. Discrete variables are described as number and percentage. Unadjusted differences between obese and patients who were nonobese with PE were compared using the chi-squared test or Student’s *t*-test as appropriate.

#### 2.5.1 Etiologic analyses

We used the binomial method to compute the 95% CIs of the observed rates of outcomes. We first compared rates of primary (all-cause death at 30 days and 6 months) and secondary outcomes, between patients who were obese (ie, BMI ≥ 30 kg/m^2^) and those who were nonobese, nonunderweight (ie, 18.5 ≤ BMI < 30 kg/m^2^) using multivariable logistic regression for 30-day outcomes and using a multivariable Cox regression model for 6-month outcomes. We used BMI as a binary covariate (obese or nonobese, nonunderweight) to reflect the pragmatic, and often clinical, approach used in bedside-risk VTE scores, in inclusion/exclusion criteria for clinical trials, and in coded datasets. We used the subdistribution hazard model for secondary outcomes, which accounts for other causes of death as competing risks [30]. We used penalized multivariable models to reduce the bias related to a low event rate. Multivariable models were adjusted for baseline characteristics, in-hospital management, and discharge treatments (known to be potential associated risk factors for mortality in the acute phase of PE) that yielded a *P* value of <.10 by univariable analysis. The potential for covariate multiple collinearities was tested using the variance inflation factor and condition number, with <10 and <30 as reference values, respectively. The full list of candidate covariates is given in Supplementary Table S2. The use of multiple imputation was not required, as the rate of missing data was <2% for all covariates (Supplementary Table S2). Results are reported as odds ratios (ORs), or hazard ratios (HRs) with associated 95% CIs. The rates of the primary outcome at 6 months are displayed by adjusted curves. We further analyzed the causal relationship between BMI and outcomes by assessing the multivariable associations between BMI, as a continuous covariate (tested with restricted cubic spline [RCS] function and 5 default knots to overcome potential issues with linearity), and outcomes (primary and secondary). To assess the robustness of the findings, we performed additional statistical event-free survival analyses, namely inverse probability of treatment weighting (IPTW). The IPTW approach aimed to adjust for confounding due to differences between the groups, assigning a weight of mean of propensity scores (PS)/PS for the obese group and (1 − mean of PS)/(1 − PS) for the nonobese group, where PS was the probability that each individual will be assigned to the obesity group. The description of the IPTW approach is provided in Supplementary Material.

#### 2.5.2 Prognostic analyses

Then, we evaluated the incremental value of adding BMI information (either BMI ≥ 30 kg/m^2^ or transformed using RCS function) on top of the ESC prognostic algorithm for the prediction of the mortality risk at 30 days [18], regardless of the causal relationship in prognostic analyses [31]. In this context, we assessed the following parameters: changes in measure of overall fit (ie, Akaike Information Criterion, Bayes Information Criterion [BIC], and Nagelkerke *R*^2^), statistical comparison of Harrell's C statistic, changes in indices of calibration (ie, Hosmer–Lemeshow parameters), predicted risk reclassification by calculating the continuous Net Reclassification Index (NRI) and integrated discrimination improvement (IDI) for the model comparisons using the obesity covariate [32].

Results were externally validated in the dataset from the RIETE registry (ClinicalTrials.gov identifier, NCT02832245). The RIETE registry is an ongoing, prospective, noninterventional international registry of consecutive patients with acute VTE, with 194 active collaborating centers across 26 countries in the Americas, Asia, and Europe. The methodology of the RIETE registry has been previously summarized elsewhere [33].

Statistical significance was defined as a 2-tailed *P* value of <.05 for all analyses. All statistical analyses were performed with SAS, version 9.4.

## 3 Results

### 3.1 Study population

A total of 2979 patients were admitted to the participating centers with a diagnosis of confirmed PE between January 2011 and July 2021. Among them, 3 patients (0.1%) were lost to follow-up, and 586 patients (19.7%) were underweight (ie, < 18.5 kg/m^2^); 876 patients (29.4%) had normal weight, 828 patients (27.8%) were overweight, and 686 patients had obesity (23.0%). Supplementary Table S3 describes the characteristics of patients characterized as underweight vs nonunderweight. The final study population comprised 2390 patients (80.3%) with a BMI of ≥18.5 kg/m^2^ (mean age, 66.9 ± 16.8 years; 1188 men [49.7%]) (Supplementary Figure S1 and Table 1). Overall, 67 patients (2.8%) had ESC-defined high-risk PE, 611 patients (25.6%) intermediate-high risk PE, 1356 patients (56.7%) intermediate-low risk PE, and 356 patients (14.9%) low-risk PE (Table 1). Follow-up was performed by a hospital visit (83.3%), review of hospitalization records (10.3%), telephone contact (5.3%), and consultation of the national death registry (1.1%).

**Table 1:** Baseline characteristics of the study population (*n* = 2390) with acute pulmonary embolism, according to BMI-defined obesity or nonobesity status.

Variables	Total (*n* = 2390)	Obesity^a^ (*n* = 686)	No obesity (*n* = 1704)	Absolute difference (95% CI)
Age (y), mean ± SD	66.9 ± 16.8	64.9 ± 15.7	67.7 ± 17.2	3.1 ± 1.5
Male, *n* (%)	1188 (49.7)	303 (44.2)	885 (51.9)	7.7 (3.2-12.1)
BMI (kg/m^2^), mean ± SD	27.6 ± 5.9	34.8 ± 5.3	24.7 ± 2.8	2.1 ± 1.3
Comorbidities, *n* (%)				
Pulmonary disease/HF	189 (7.9)	68 (9.9)	121 (7.1)	2.8 (0.4-5.2)
Previous stroke	118 (4.9)	32 (4.7)	86 (5.0)	0.3 (−1.6 to 02.2)
Active cancer^b^	452 (18.9)	89 (12.9)	363 (21.3)	8.4 (4.9-11.9)
Previous VTE	557 (23.3)	203 (29.6)	354 (20.8)	8.8 (5.0-12.5)
Previous bleeding	15 (2.3)	11 (2.4)	4 (2.2)	0.2 (−1.1 to 1.5)
Transient or reversible factors of VTE	576 (24.1)	181 (31.4)	395 (21.2)	10.2 (6.4-14.0)
No identifiable risk factor (unprovoked PE)	1814 (75.9)	505 (73.6)	1,09 (76.8)	3.2 (−0.6 to 7.0)
Associated DVT	156 (25.1)	298 (30.5)	679 (39.8)	9.3 (5.0-13.6)
Clinical characteristics				
Syncope at presentation, *n* (%)	156 (6.5)	47 (6.8)	109 (6.4)	0.4 (−1.8 to 2.6)
HR at admission (bpm), mean ± SD	89.7 ± 18.4	90.9 ± 17.9	89.2 ± 18.7	1.1 ± 1.9
SBP at admission (mm Hg) mean ± SD	186.6 ± 23.4	141.0 ± 23.5	137.7 ± 23.3	2.2 ± 1.8
SaO_2_ (%), mean ± SD	993.4 ± 5.8	93.1 ± 7.2	93.7 ± 5.1	0
Biological data				
Positive troponin, *n* (%)	84 (10.5)	273 (39.8)	656 (38.5)	1.3 (−3.0 to 5.6)
Hemoglobin at admission (g/dL), mean ± SD	13.3 ± 2.0	13.5 ± 2.0	13.2 ± 2.0	—
eGFR at admission (mmol/L), mean ± SD	82.1 ± 33.3	77.9 ± 34.5	83.7 ± 32.7	—
Echocardiography data				
RV dysfunction^c^, *n* (%)	973 (40.7)	289 (42.1)	675 (39.6)	2.5 (−1.8 to 6.8)
sPESI (points, Q1–Q3)	2 (1-3)	2 (1-3)	2 (1-3)	—
ESC-defined risk stratification of index PE, *n* (%)				
Low risk	356 (14.9)	120 (17.5)	236 (13.8)	3.7 (0.5-6.8)
Intermediate-low risk	1356 (56.7)	354 (51.6)	1002 (58.8)	7.2 (2.8-11.6)
Intermediate-high risk	611 (25.6)	193 (28.1)	418 (24.5)	3.6 (−0.2 to 7.5)
High risk	67 (2.8)	19 (2.8)	48 (2.8)	0 (−1.5 to 1.5)

BMI, body mass index; DVT, deep vein thrombosis; eGFR, estimated glomerular filtration rate; ESC, The European Society of Cardiology; HF, heart failure; HR, heart rate; PE, pulmonary embolism; RV, right ventricular; SaO_2_, arterial oxyhemoglobin saturation; SBP, systolic blood pressure; sPESI, simplified Pulmonary Embolism Severity Index; VTE, venous thromboembolic event.

a BMI ≥ 30 kg/m^2^.

b Cancer is considered active when at least 1 of the following 3 conditions is met: (1) the patient has received a potentially noncurative treatment of his cancer (case in particular of so-called palliative chemotherapy); (2) the evolution shows recurrence or progression of the cancer under treatment; and (3) the cancer treatment is ongoing.

c RV dysfunction was defined by at least 1 of the following parameter: a right ventricle/left ventricle diameter ratio > 1.0 on echocardiography or computed tomography scan, flattened intraventricular septum on echography, peak systolic gradient at the tricuspid valve of >30 mmHg, or a tricuspid annular plane systolic excursion of <16.0 mm.

### 3.2 Patient characteristics

The mean BMI in the study population was 27.6 ± 5.9 kg/m^2^ (median, 26.6 kg/m^2^; Q1-Q3, 23.7-30.7 kg/m^2^). In total, 686 (28.7%) patients had BMI of ≥30 kg/m^2^ (obese group), and 1704 (71.3%) were not obese. Overall, patients in the obese group were younger, were more frequently women, and less frequently had active cancer. PE severity based on cardiac troponin, right ventricular dysfunction, simplified Pulmonary Embolism Severity Index score, and ESC-defined early risk stratification did not differ significantly between obese and patients who were nonobese (Table 1). Patients in the obesity group were more frequently treated with unfractionated heparin at admission and more frequently received systemic thrombolysis (7.6% vs 3.3%) compared with the nonobese group. Patients with obesity were less frequently treated with low molecular weight heparin and more frequently with a direct oral anticoagulant at discharge. Catheter-directed therapy was not performed in the participating centers during the study period. The adjusted length of hospital stay did not differ between groups (Supplementary Table S4).

### 3.3 Outcomes: etiologic analysis

Supplementary Table S5 summarizes observed rates, incidence rates per patient-year, and risk estimates of clinical outcomes between obese and nonobese patients. During the first 30 days of follow-up, there were 122 all-cause deaths (5.1%), 54 major bleeding episodes (2.3%), and 36 CRNM bleeding events (1.5%). At 6 months, we observed 333 deaths (13.9%), 101 major bleeding (4.2%) and 82 CRNM bleeding events (3.4%), 37 recurrent VTE (1.5%), 14 acute MI (0.6%), and 17 strokes (0.7%). Cause of death was predominantly cancer (39.3% at 30 days and 52.8% at 6 months) (Supplementary Table S6). Supplementary Table S7 summarizes the causes of major bleeding. Supplementary Table S8 displays covariates associated with the occurrence of outcomes in univariable analyses.

**Figure 1:**
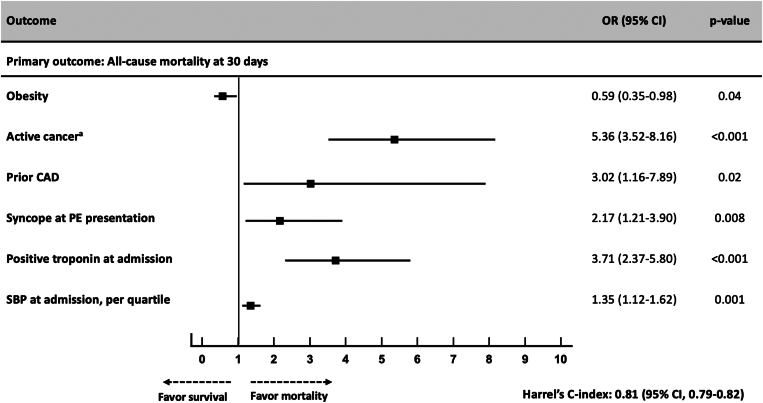
Clinical factors independently associated with the 30-day all-cause mortality after acute pulmonary embolism. Obesity is defined by a body mass index of ≥30 kg/m^2^ according to the WHO definition. ^a^Cancer is considered active when at least one of the following 3 conditions is met: (1) the patient has received a potentially noncurative treatment for cancer (case in particular of so-called palliative chemotherapy); (2) the evolution shows recurrence or progression of the cancer under treatment; and (3) cancer treatment is ongoing. Covariates used for the multivariate adjustment are given in Supplementary Table S7. CAD, coronary artery disease; PE, pulmonary embolism; SBP, systolic blood pressure; OR, odds ratio.

**Figure 2:**
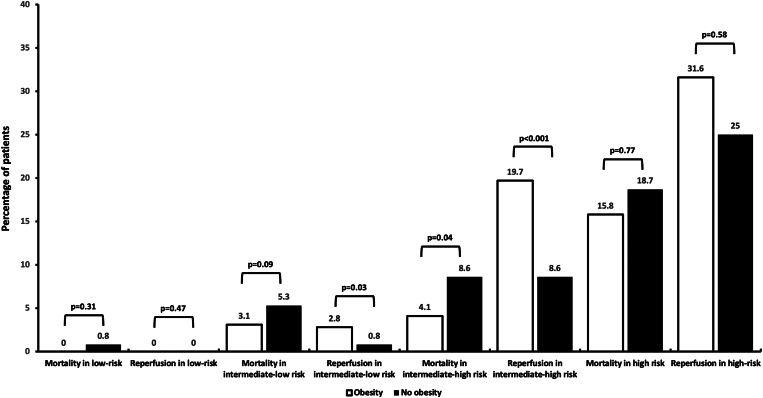
Cumulative frequencies of acute pulmonary embolism all-cause death at 30 days and associated pulmonary reperfusion strategies according to the European Society of Cardiology (ESC) prognosis risk category (*P* values, unadjusted comparison).

**Figure 3:**
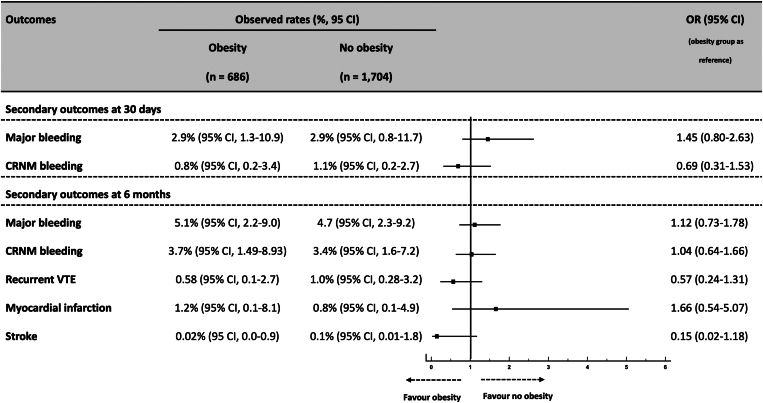
Secondary outcomes at 30 days and 6 months between patients with and without obesity after acute pulmonary embolism. Obesity is defined by a body mass index of ≥30 kg/m^2^ according to the WHO definition. CRNM, clinically relevant nonmajor; OR, odds ratio; VTE, venous thromboembolism.

**Figure 4:**
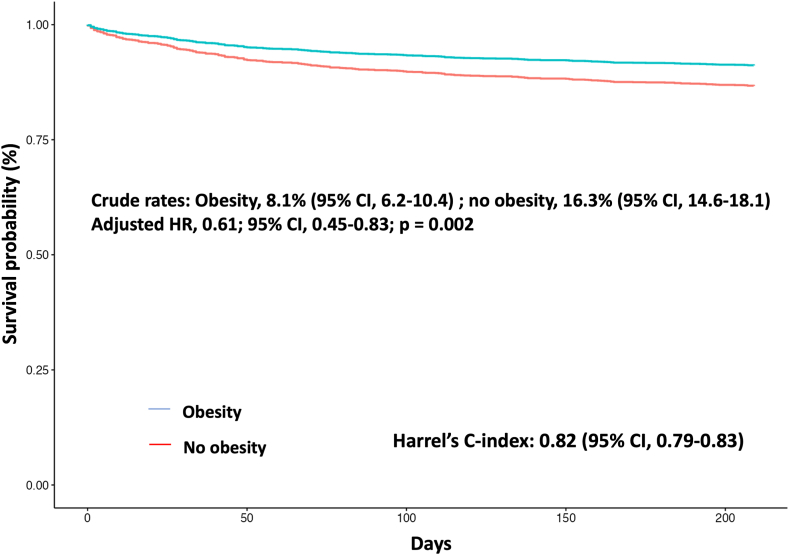
Cox model–derived adjusted curves for the primary outcome, all-cause death at 6 months, between patients with and without obesity after acute pulmonary embolism. Obesity is defined by a body mass index of ≥30 kg/m^2^ according to the WHO definition. Covariates used for the multivariate adjustment are given in Supplementary Table S7. HR, hazard ratio.

The absolute rate of the primary outcome at 30 days (ie, all-cause mortality) was 1.9% (95% CI, 0.7-4.8) in patients with obesity and 3.5% (95% CI, 1.2-7.1) in those without obesity (absolute difference, 1.6%; 95% CI, 0.1-3.1), yielding an OR of 0.59 (95% CI, 0.35-0.98) for patients with WHO-defined obesity (Figure 1). Figure 2 displays the crude 30-day mortality rates across ESC-defined risk categories and the associated rates of thrombolysis used in each category. The observed mortality rate in intermediate-high risk PE was significantly lower in patients with obesity than that in those without (absolute rate, 4.1% [95% CI, 1.8-7.9] vs 8.6% [95% CI, 6.1-11.7]) with a higher rate of thrombolytics used in the obese group for this PE risk category. Adjusted rates of the secondary bleeding outcomes at 30 days were similar between groups (Figure 3). The absolute rates of all-cause mortality at 6 months were significantly lower in patients with obesity than those in patients who were nonobese (4.5% vs 8.7%; HR, 0.61; 95% CI, 0.45-0.83) (Figure 4). The performance of the 6-month all-cause death multivariable model is displayed in Supplementary Figure S2 (mean Harrell C index, 0.79; Hosmer–Lemeshow *P* = .09). The absolute rates of other outcomes at 6 months did not differ between obesity and nonobesity groups (Figure 3).

BMI, as an RCS-transformed continuous variable, was independently associated with mortality at 30 days and 6 months. These associations were nonlinear. Analysis of RCS curve shapes suggested that a BMI lower than 30 kg/m^2^ was continuously associated with increased mortality hazards at 30 days and 6 months (HR >1.0), whereas mortality rates decreased beyond a BMI ≥ 30 kg/m^2^ (Supplementary Figure S3). RCS-transformed BMI was not associated with secondary outcomes (Supplementary Figure S4).

Among the study population, 44 patients (1.8%) had acute COVID-19 infection during the study period. There was no difference in mortality at 30 days between the 14 patients with COVID-19 and obesity, and the 30 patients with COVID-19 and no obesity (9.3% vs 7.5%; univariate *P* = .32).

### 3.4 Improved risk stratification when adding obesity: prognostic analysis

The addition of the obesity information on top of the ESC model improved the global model fit with a higher Nagelkerke *R*^2^ and lower Akaike Information Criterion and Bayes Information Criterion. The model had better discriminatory capacity with a significant increase in Harrell's C index (0.636 vs 0.657; *P* = .007) and better calibration with an increase in Hosmer–Lemeshow parameters. The IDI and NRI increased significantly when adding obesity as an additional covariate in the ESC model, yielding 10.3% of predicted 30-day death reclassification based on the observed mortality rates with the ESC model as reference (Table 2). A similar improvement in predicted risk was observed with the model comparisons using BMI as a continuous covariate in addition to the ESC prognostic algorithm (Supplementary Table S9).

**Table 2:** Overall model fit, discrimination, calibration indices, and predicted risk reclassification when obesity (BMI ≥ 30 kg/m^2^) is added or not added to the European Society of Cardiology (ESC) model for the prediction of the 30-day all-cause death after acute pulmonary embolism in the study population and in the RIETE cohort for external validation.

Model information	Study population (*n* =2390)		External validation (*n* = 35,796)	
	ESC model with obesity^a^		ESC model with obesity^a^	
	No	Yes	No	Yes
Models (OR, 95% CI)				
ESC model	2.18 (1.69-2.81)	2.20 (1.71-2.84)	1.85 (1.74-1.96)	1.83 (1.73-1.94)
ESC model with obesity^a^	—	0.51 (0.32-0.83)	—	0.57 (0.50-0.64)
Overall model fit				
Bayes information criteria	942.7	942.2	12144.8	12070.5
Akaike information criteria	931.2	924.2	12127.9	12045.1
Nagelkerke *R*^2^ (%)	1.6	0.05	0.04	0.05
Discrimination				
Harrell C index	0.636	0.657^b^	0.654	0.673^c^
Calibration				
*P* (Hosmer–Lemeshow)	.02	.13	<.001	.09
Risk reclassification between ESC model and ESC model with obesity^a^				
IDI	3.7% (95% CI, 0.8-6.7; *P* = .01)		2.5% (95% CI, 1.9-3.2; *P* < .01)	
Continuous NRI	22.4% (95% CI, 8.4-36.6; *P* = .01)		23.1% (95% CI, 18.8-27.3; *P* < .001)	
Reclassification rate^d^ (%)	10.3		8.9	

ESC, European Society of Cardiology; IDI, integrated discrimination improvement; NRI, net reclassification improvement; OR, odds ratio.

a Defined by a body mass index of ≥30 kg/m^2^.

b Difference in Harrell C indices with *P* = .007.

c Difference in Harrell C indices with *P* < .001.

d Reclassification based on the observed mortality rates with the ESC model as reference.

### 3.5 Sensitivity analyses

For the IPTW analyses, we confirmed that the standardized mean differences were <0.1 for all covariates (Supplementary Figure S5). Using the IPTW to correct differences between patients with and without obesity, we observed a lower rate of death at 30 days and at 6 months (OR, 0.66; 95% CI, 0.45-0.97; and HR, 0.72; 95% CI, 0.57-0.91, respectively) after acute PE in obese patients.

### 3.6 External validation

**Figure 5:**
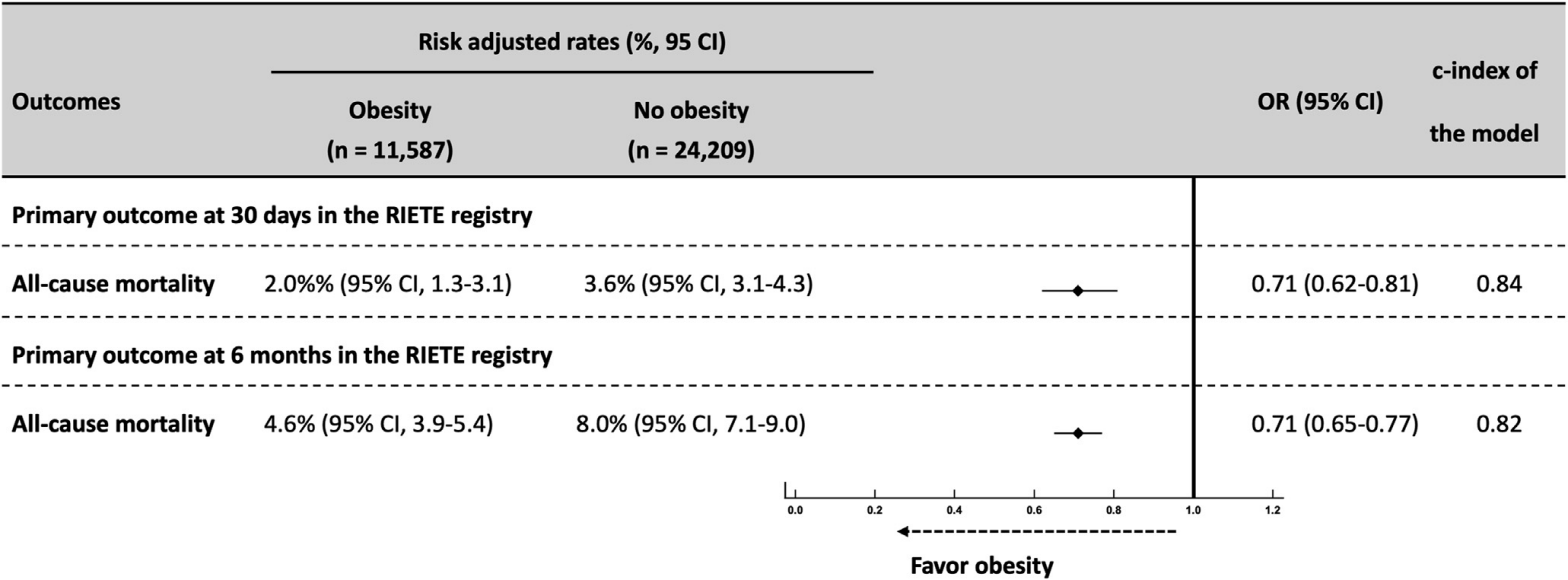
External validation of the primary outcomes, all-cause death at 30 days and 6 months, between patients with and without obesity after acute pulmonary embolism recorded in the RIETE registry (*n* = 35,796). OR, odds ratio.

Among the 35,796 patients with PE and BMI of ≥18.5 kg/m^2^ (mean age, 66.0 ± 16.8 years; males, 48.3%) included in the RIETE validation cohort between January 2001 and December 2021, 11,587 patients were obese (32.3%) and 24,209 (67.7%) were nonobese, nonunderweight. The ESC prognostic risk score classified 19,762 patients (38.8%) at low risk of mortality at 30 days, 21,184 patients (41.6%) at intermediate-low risk, 8310 patients at intermediate-high risk, and 1612 patients (3.7%) at high-risk (Supplementary Table S10). The rate of 30-day all-cause death in the RIETE registry was 4.4% (95% CI, 4.2-4.6; *n* = 1587 patients). The adjusted mortality rates were significantly lower in patients with WHO-defined obesity when compared with those with BMI between 18.5 and 30 kg/m^2^ (OR, 0.71; 95% CI, 0.62-0.81, for 30-day mortality, and OR, 0.71; 95% CI, 0.65-0.77, for 6-month mortality) (etiologic analysis) (Figure 5). Overall global fit and calibration capacities were better when the obesity information was added to ESC model in the RIETE population. The Harrell C index increased from 0.654 with the ESC model alone to 0.673 with the ESC model including obesity (*P* = .001). Reclassification parameters including the IDI and NRI were significantly improved in the ESC model with the obesity covariate (*P* < .01 and *P* < .001, respectively) (prognostic analysis) (Table 2). Predicted risk also showed a similar improvement when BMI was included as a continuous covariate in addition to the ESC prognostic algorithm (Supplementary Table S9).

## 4 Discussion

In this multicenter cohort study, the etiologic analysis showed lower early- and mid-term mortality rates in patients with BMI-defined obesity after PE compared with patients who were nonobese, nonunderweight, independently of other covariates, including PE severity, presence of cancer, or use of reperfusion therapies. Patients with obesity received different anti-thrombotic strategies during the acute phase (eg, higher rate of systemic thrombolysis), which could at least partially explain the lower 30-day mortality observed, especially in the intermediate-high risk PE category. The addition of the BMI parameter on the top of the ESC prognostic algorithm significantly improved early mortality risk prediction in the prognostic analyses. Results were confirmed in over 35,000 patients from the multinational RIETE registry.

Several studies have previously reported an obesity paradox, whereby patients with obesity have better prognosis and reduced mortality than their leaner counterparts in noncardiovascular (eg, chronic kidney disease, infection) [34,35], and cardiovascular settings (coronary artery disease, heart failure) [36,37]. Our analysis corroborates prior data by showing similar findings in PE. Barba et al. [12] first reported a survival benefit in patients with obesity after 3 months of therapy among 10,114 patients with VTE (OR, 0.5; 95% CI, 0.4-0.6). Data from the Global Anticoagulant Registry in the FIELD-Venous Thrombo-Embolism registry recently showed that the risk of all-cause mortality was lower in overweight and patients with obesity than in those with normal BMI (adjusted HR, 0.75; 95% CI, 0.63-0.89, and 0.59; 95% CI, 0.49-0.72, respectively) with 24 months of follow-up after acute PE [11]. Two large nationwide inpatient samples including 2,237,660 and 345,831 patients with PE, respectively, described a lower in-hospital mortality rate in the obesity group (unadjusted OR, 0.45; 95% CI, 0.44-0.46, in the US registry, and adjusted OR, 0.74; 95% CI, 0.71-0.77, in the German registry) [13,14]. Finally, the risk of death during anticoagulation (around 6 months) was about one-third lower in patients with morbid obesity (ie, BMI ≥ 40 kg/m^2^) than that in those with normal weight in the RIETE registry (*n* = 16,490) [10].

The reduction of mortality in the obesity group was more pronounced for patients with intermediate-high risk PE, a category in which a higher rate of systemic thrombolysis was observed in parallel. Bleeding complications are the most feared consequence of thrombolytic-based therapy. Major bleeding occurs in 9.9% after the administration of systemic thrombolysis (vs 3.6% with heparin), with an associated intracranial or fatal hemorrhage rate of 1.7% (vs 0.3% with heparin) [38]. Lower body weight was previously identified as a risk factor for thrombolysis-related bleeding in the PE and in the non-PE settings (ie, MI) [39,40]. In a case–control study, each 10 kg below 100 kg was independently associated with bleeding in PE patients treated with thrombolysis (OR 1.18; 95% CI, 1.01-1.37) [40]. Overall, and similar to other reports [13], our data underline clinician concerns about the bleeding risk associated with thrombolytic therapy and lower body weight [41].

Our multivariate and IPTW analyses in the etiology studies strongly suggest that the survival benefit in patients with obesity is independently related to the BMI itself, rather than associated comorbidities or reperfusion therapies. A variety of mechanisms have been proposed to explain the obesity paradox in patients with cardiovascular disease (CVD) and critical illness [42], with the identification of metabolically healthy obese phenotypes [17]. Patients with CVD who are overweight/obese but with a high proportion of abdominal fat or a high muscle mass (ie, lean mass index) appear to have better prognosis, even if they have increased body fat content, when compared with patients with low body fat and low lean mass [43]. Obesity is often associated with obstructive sleep apnea, which may cause intermittent hypoxia [44]. This in turn could lead to hypoxic preconditioning, thereby improving the organs’ response to ischemia and reducing ischemia-reperfusion injuries [45]. Finally, a meta-analysis of 10 studies examining the joint associations of cardiorespiratory fitness and BMI on mortality concluded that the obesity paradox does not apply among cardiorespiratory-fit individuals, whereas among cardiorespiratory unfit individuals, overweight and obese individuals had a more than 2-fold higher risk for mortality, compared with their normal-weight, cardiorespiratory-fit counterparts [46]. Nevertheless, a meta-analysis of 6 studies including 15,923 patients with CVD directly compared the mortality risk of waist circumference (WC) or waist-to-hip ratio as indices of central obesity and BMI as a parameter of total adiposity [47]. The relationship between BMI and mortality represented a negative association reflecting the typical obesity paradox pattern. However, central obesity was positively associated with mortality, even among patients with an apparently normal BMI. In this context, some authors have proposed to recognize a BMI paradox in lieu of an obesity paradox [17].

In this analysis, we demonstrated that incorporating obesity parameters into the ESC risk algorithm improves 30-day mortality prediction, similar to previous findings with lactate levels and renal function [48,49]. In this context, some authors have highlighted limitations of the current PE risk stratification [50]. Based on these findings, additional parameters, including obesity, should likely be integrated into future risk models, potentially leveraging artificial intelligence for enhanced prognostic accuracy [51].

The main limitation of this study is its inability to provide information about other biometric parameters known to be linked to obesity evaluation, especially those analyzing central obesity (ie, WC and waist-to-hip ratio), as opposed to assessing total adiposity using the BMI. It is also possible that other potential explanatory comorbidities exist that we did not include in the multivariate adjustment. By excluding underweight patients from the nonobese cohort, this may introduce some potential for selection bias due to the factors associated with low body weight like cancer, malnutrition, or chronic illness. Nevertheless, underweight patients are likely to be phenotypically different from nonunderweight individuals, and these differences would have had the potential to directly impact the outcome of interest, despite appropriate statistical adjustment, justifying their exclusion [13]. Despite adjustments for potential confounders, including sensitivity analyses using the IPTW approach, residual confounding cannot be excluded, particularly given differences in treatment choices and the high prevalence of cancer-related mortality, which may have influenced both PE occurrence and survival outcomes [52]. Further, while we could not determine whether patients with obesity were more likely to die outside of the hospital or before diagnosis of PE, we reviewed our data on 12 patients who presented with cardiac arrest. Assuming that all experienced fatal PE, this subset represented a very small proportion of the overall study population (0.5%) and would be unlikely to change the overall results of our mortality findings. Finally, race/ethnicity data were not collected in this study because this is not authorized by currently French legislation governing research. The strengths of our analysis include the prospective inclusion of our cohort population, the high rate of complete follow-up, the blinded, independent adjudication of clinical outcomes to reduce ascertainment bias, and the appropriate statistical approach. Moreover, data were externally validated in the large (>35,000 patients) worldwide RIETE registry, enabling generalization of our results. In addition, contrary to other reports [13], the rate of obesity in our cohort (ie, 23% vs 8.6% in a previous report on VTE) [13] was similar to the actual prevalence of this metabolic disorder [53], ensuring the generalization of the present results in a population of European Caucasian descent.

## 5 Conclusion

We present evidence indicating lower early- and mid-term mortality after acute PE in patients classified as obese based on BMI, compared with that in patients who were nonobese, nonunderweight. Additional studies are needed to assess the prognostic significance of central obesity as opposed to the total adiposity as indicated by BMI. In the meantime, BMI, which is easily available, may have utility when incorporated into algorithms or scoring systems for predicting early mortality following acute PE.

## References

[1] Finucane M.M., Stevens G.A., Cowan M.J., Danaei G., Lin J.K., Paciorek C.J. (2011). Global Burden of Metabolic Risk Factors of Chronic Diseases Collaborating Group (Body Mass Index). National, regional, and global trends in body-mass index since 1980: systematic analysis of health examination surveys and epidemiological studies with 960 country-years and 9.1 million participants. Lancet.

[2] Ward Z.J., Willett W.C., Hu F.B., Pacheco L.S., Long M.W., Gortmaker S.L. (2022). Excess mortality associated with elevated body weight in the USA by state and demographic subgroup: a modelling study. EClinicalMedicine.

[3] Gregson J., Kaptoge S., Bolton T., Pennells L., Willeit P., Burgess S. (2019). Emerging Risk Factors Collaboration. Cardiovascular risk factors associated with venous thromboembolism. JAMA Cardiol.

[4] Rahmani J., Haghighian Roudsari A., Bawadi H., Thompson J., Khalooei Fard R., Clark C. (2020). Relationship between body mass index, risk of venous thromboembolism and pulmonary embolism: a systematic review and dose-response meta-analysis of cohort studies among four million participants. Thromb Res.

[5] Eichinger S., Hron G., Bialonczyk C., Hirschl M., Minar E., Wagner O. (2008). Overweight, obesity, and the risk of recurrent venous thromboembolism. Arch Intern Med.

[6] Joshy G., Korda R.J., Attia J., Liu B., Bauman A.E., Banks E. (2014). Body mass index and incident hospitalisation for cardiovascular disease in 158 546 participants from the 45 and Up Study. Int J Obes (Lond).

[7] Klovaite J., Benn M., Nordestgaard B.G. (2015). Obesity as a causal risk factor for deep venous thrombosis: a Mendelian randomization study. J Intern Med.

[8] Lindstrom S., Germain M., Crous-Bou M., Smith E.N., Morange P.E., van Hylckama Vlieg A., INVENT Consortium (2017). Assessing the causal relationship between obesity and venous thromboembolism through a Mendelian randomization study. Hum Genet.

[9] Lilja L., Bygdell M., Martikainen J., Rosengren A., Kindblom J.M., Ohlsson C. (2023). Overweight in childhood and young adulthood increases the risk for adult thromboembolic events. J Intern Med.

[10] Giorgi-Pierfranceschi M., Lopez-Nunez J.J., Monreal M., Cattabiani C., Lodigiani C., Di Micco P. (2020). RIETE Researchers. Morbid obesity and mortality in patients with VTE: findings from real-life clinical practice. Chest.

[11] Weitz J.I., Farjat A.E., Ageno W., Turpie A.G.G., Haas S., Goto S., GARFIELD-VTE Investigators (2021). Influence of body mass index on clinical outcomes in venous thromboembolism: insights from GARFIELD-VTE. J Thromb Haemost.

[12] Barba R., Zapatero A., Losa J.E., Valdes V., Todoli J.A., Di Micco P., Riete Investigators (2008). Body mass index and mortality in patients with acute venous thromboembolism: findings from the RIETE registry. J Thromb Haemost.

[13] Keller K., Hobohm L., Munzel T., Ostad M.A., Espinola-Klein C., Lavie C.J. (2019). Survival benefit of obese patients with pulmonary embolism. Mayo Clin Proc.

[14] Stein P.D., Matta F., Goldman J. (2011). Obesity and pulmonary embolism: the mounting evidence of risk and the mortality paradox. Thromb Res.

[15] Alkhalfan F., Bukhari S., Rosenzveig A., Moudgal R., Khan S.Z., Ghoweba M. (2024). The obesity mortality paradox in patients with pulmonary embolism: insights from a tertiary care center. J Clin Med.

[16] Yang G., De Staercke C., Hooper W.C. (2012). The effects of obesity on venous thromboembolism: a review. Open J Prev Med.

[17] Kim S.H., Despres J.P., Koh K.K. (2016). Obesity and cardiovascular disease: friend or foe?. Eur Heart J.

[18] Konstantinides S.V., Meyer G., Becattini C., Bueno H., Geersing G.J., Harjola V.P., ESC Scientific Document Group (2020). 2019 ESC Guidelines for the diagnosis and management of acute pulmonary embolism developed in collaboration with the European Respiratory Society (ERS). Eur Heart J.

[19] Chopard R., Albertsen I.E., Ecarnot F., Guth S., Besutti M., Falvo N. (2022). Extended anticoagulation after pulmonary embolism: a multicenter observational cohort analysis. J Am Heart Assoc.

[20] PIOPED Investigators (1990). Value of the ventilation/perfusion scan in acute pulmonary embolism. Results of the prospective investigation of pulmonary embolism diagnosis (PIOPED). JAMA.

[21] Remy-Jardin M., Remy J., Wattinne L., Giraud F. (1992). Central pulmonary thromboembolism: diagnosis with spiral volumetric CT with the single-breath-hold technique—comparison with pulmonary angiography. Radiology.

[22] Jaff M.R., McMurtry M.S., Archer S.L., Cushman M., Goldenberg N., Goldhaber S.Z., American Heart Association Council on Cardiopulmonary, Critical Care, Perioperative and Resuscitation; American Heart Association Council on Peripheral Vascular Disease (2011). American Heart Association Council on Arteriosclerosis, Thrombosis and Vascular Biology. Management of massive and submassive pulmonary embolism, iliofemoral deep vein thrombosis, and chronic thromboembolic pulmonary hypertension: a scientific statement from the American Heart Association. Circulation.

[23] Kearon C., Akl E.A., Ornelas J., Blaivas A., Jimenez D., Bounameaux H. (2016). Antithrombotic therapy for VTE disease: CHEST guideline and expert panel report. Chest.

[24] Konstantinides S.V. (2014). 2014 ESC Guidelines on the diagnosis and management of acute pulmonary embolism. Eur Heart J.

[25] (2000). Obesity: preventing and managing the global epidemic. Report of a WHO consultation. World Health Organ Tech Rep Ser.

[26] Thygesen K., Alpert J.S., Jaffe A.S., Chaitman B.R., Bax J.J., Morrow D.A., Executive Group on behalf of the Joint European Society of Cardiology (ESC)/American College of Cardiology (ACC)/American Heart Association (AHA)/World Heart Federation (WHF) Task Force for the Universal Definition of Myocardial Infarction (2018). Fourth universal definition of myocardial infarction (2018). Circulation.

[27] Ageno W., Haas S., Weitz J.I., Goldhaber S.Z., Turpie A.G.G., Goto S., Angchaisuksiri P., Nielsen J.D., Kayani G., Pieper K.S., Schellong S., Bounameaux H., Mantovani L.G., Prandoni P., Kakkar A.K., investigators G-V (2019). Characteristics and management of patients with venous thromboembolism: The GARFIELD-VTE Registry. Thromb Haemost.

[28] Althouse A.D., Below J.E., Claggett B.L., Cox N.J., de Lemos J.A., Deo R.C. (2021). Recommendations for statistical reporting in cardiovascular medicine: a special report from the American Heart Association. Circulation.

[29] Collins G.S., Reitsma J.B., Altman D.G., Moons K.G. (2015). Transparent reporting of a multivariable prediction model for individual prognosis or diagnosis (TRIPOD): the TRIPOD Statement. BMC Med.

[30] Austin P.C., Fine J.P. (2017). Practical recommendations for reporting Fine-Gray model analyses for competing risk data. Stat Med.

[31] Nemeth B., Smeets M.J.R., Cannegieter S.C., van Smeden M. (2024). Tutorial: dos and don’ts in clinical prediction research for venous thromboembolism. Res Pract Thromb Haemost.

[32] Cook N.R., Paynter N.P. (2011). Performance of reclassification statistics in comparing risk prediction models. Biom J.

[33] Bikdeli B., Jimenez D., Hawkins M., Ortiz S., Prandoni P., Brenner B., RIETE Investigators (2018). Rationale, Design and Methodology of the Computerized Registry of Patients with Venous Thromboembolism (RIETE). Thromb Haemost.

[34] Nie W., Zhang Y., Jee S.H., Jung K.J., Li B., Xiu Q. (2014). Obesity survival paradox in pneumonia: a meta-analysis. BMC Med.

[35] Jialin W., Yi Z., Weijie Y. (2012). Relationship between body mass index and mortality in hemodialysis patients: a meta-analysis. Nephron Clin Pract.

[36] Shah R., Gayat E., Januzzi J.L., Sato N., Cohen-Solal A., diSomma S., GREAT (Global Research on Acute Conditions Team) Network (2014). Body mass index and mortality in acutely decompensated heart failure across the world: a global obesity paradox. J Am Coll Cardiol.

[37] Wang Z.J., Zhou Y.J., Galper B.Z., Gao F., Yeh R.W., Mauri L. (2015). Association of body mass index with mortality and cardiovascular events for patients with coronary artery disease: a systematic review and meta-analysis. Heart.

[38] Marti C., John G., Konstantinides S., Combescure C., Sanchez O., Lankeit M. (2015). Systemic thrombolytic therapy for acute pulmonary embolism: a systematic review and meta-analysis. Eur Heart J.

[39] Berkowitz S.D., Granger C.B., Pieper K.S., Lee K.L., Gore J.M., Simoons M., Incidence and predictors of bleeding after contemporary thrombolytic therapy for myocardial infarction. The Global Utilization of Streptokinase and Tissue Plasminogen activator for Occluded coronary arteries (GUSTO) I Investigators (1997). Circulation.

[40] Curtis G.M., Lam S.W., Reddy A.J., Bauer S.R. (2014). Risk factors associated with bleeding after alteplase administration for pulmonary embolism: a case-control study. Pharmacotherapy.

[41] Chopard R., Campia U., Jering K.S., Almarzooq Z.I., Snyder J.E., Rizzo S. (2023). Guideline adherence and clinical outcomes of patients with acute pulmonary embolism evaluated by a multidisciplinary response team at a quaternary care center. Thromb Res.

[42] Zhou D., Wang C., Lin Q., Li T. (2022). The obesity paradox for survivors of critically ill patients. Crit Care.

[43] Lavie C.J., De Schutter A., Patel D.A., Romero-Corral A., Artham S.M., Milani R.V. (2012). Body composition and survival in stable coronary heart disease: impact of lean mass index and body fat in the “obesity paradox.”. J Am Coll Cardiol.

[44] Tai J.E., Phillips C.L., Yee B.J., Grunstein R.R. (2024). Obstructive sleep apnoea in obesity: a review. Clin Obes.

[45] Sharafkhaneh A., Agrawal R., Nambi V., BaHammam A., Razjouyan J. (2023). Obesity paradox or hypoxia preconditioning: how obstructive sleep apnea modifies the Obesity-MI relationship. Sleep Med.

[46] Barry V.W., Baruth M., Beets M.W., Durstine J.L., Liu J., Blair S.N. (2014). Fitness vs. fatness on all-cause mortality: a meta-analysis. Prog Cardiovasc Dis.

[47] Coutinho T., Goel K., Correa de Sa D., Kragelund C., Kanaya A.M., Zeller M. (2011). Central obesity and survival in subjects with coronary artery disease: a systematic review of the literature and collaborative analysis with individual subject data. J Am Coll Cardiol.

[48] Ebner M., Sentler C., Harjola V.P., Bueno H., Lerchbaumer M.H., Hasenfuss G. (2021). Outcome of patients with different clinical presentations of high-risk pulmonary embolism. Eur Heart J Acute Cardiovasc Care.

[49] Chopard R., Jimenez D., Serzian G., Ecarnot F., Falvo N., Kalbacher E. (2021). Renal dysfunction improves risk stratification and may call for a change in the management of intermediate- and high-risk acute pulmonary embolism: results from a multicenter cohort study with external validation. Crit Care.

[50] Jimenez D., Tapson V., Yusen R.D., Becattini C., Moores L., Barnes G.D. (2023). Revised paradigm for acute pulmonary embolism prognostication and treatment. Am J Respir Crit Care Med.

[51] Pfob A., Lu S.C., Sidey-Gibbons C. (2022). Machine learning in medicine: a practical introduction to techniques for data pre-processing, hyperparameter tuning, and model comparison. BMC Med Res Methodol.

[52] Prada-Ramallal G., Takkouche B., Figueiras A. (2019). Bias in pharmacoepidemiologic studies using secondary health care databases: a scoping review. BMC Med Res Methodol.

[53] Ng M., Fleming T., Robinson M., Thomson B., Graetz N., Margono C. (2014). Global, regional, and national prevalence of overweight and obesity in children and adults during 1980-2013: a systematic analysis for the Global Burden of Disease Study 2013. Lancet.

