# Supplementary material for: Lower early mortality and risk prediction improvement of obesity after acute pulmonary embolism: results from a multicenter cohort analysis with external validation
Source: Res Pract Thromb Haemost. 2025 Feb 28;9(2):102718. doi: 10.1016/j.rpth.2025.102718 (PMC11992428; doi:10.1016/j.rpth.2025.102718)
Supplement: Supplementary Material [file mmc1.docx]

**Supplemental material**

**Lower early mortality and risk prediction improvement of obesity after acute pulmonary embolism: Results from a multicenter cohort analysis with external validation**

**Definition of outcomes:**

Causes of death were classified according to the International Society on Thrombosis and Haemostasis (ISTH) guidelines.^1^

-Death was classified as related to VTE, related to cancer, related to bleeding, or related to non-cancer, Related to other cause. VTE was considered as the cause of death if there was objective documentation or if death could not be attributed to another documented cause and PE could not be ruled out.^1^

Bleeding event were classified according to the ISTH guidelines:^2^

-Major bleeding was defined according to the ISTH criteria, namely: (1) fatal bleeding and/or (2) symptomatic bleeding in a critical area or organ (intracranial, intraspinal, intraocular, retroperitoneal, intra-articular or pericardial, or intramuscular with compartment syndrome), and/or (3) bleeding causing a fall in hemoglobin level of 20 g/L (1.24 mmol/L) or more, or leading to transfusion of two or more units of whole blood or red cells.^2^

-Clinically relevant non-major bleeding was acute clinically overt bleeding that consists of: any bleeding compromising hemodynamics; any bleeding leading to hospitalization; subcutaneous hematoma larger than 25 cm^2^, or 100 cm^2^ if there was a traumatic cause; intramuscular hematoma documented by ultrasonography; epistaxis that lasted for more than 5 minutes, was repetitive (i.e., two or more episodes of bleeding more extensive than spots on a handkerchief within 24 hours), or led to an intervention (e.g., packing or electrocoagulation); gingival bleeding occurring spontaneously (i.e., unrelated to eating or tooth brushing) or lasting for more than 5 minutes; hematuria that was macroscopic and was spontaneous or lasted for more than 24 hours after instrumentation (e.g., catheter placement or surgery) of the urogenital tract; macroscopic gastrointestinal hemorrhage, including at least one episode of rectal blood loss, if more than a few spots on toilet paper; hemoptysis, if more than a few speckles in the sputum and not occurring within the context of PE; or any other bleeding type considered to have clinical consequences for a patient such as medical intervention, the need for unscheduled contact (visit or telephone call) with a physician, or temporary cessation of a study drug, or associated with pain or impairment of activities of daily life.^2^

-Recurrent venous thromboembolism included fatal and nonfatal pulmonary embolism and deep-vein thrombosis. Recurrent VTE was defined as presence of (1) symptoms suggesting pulmonary embolism, and new defects seen on computed tomography pulmonary angiogram or ventilation-perfusion scan; or (2) diagnosis of deep vein thrombosis on compression ultrasonography.

-Acute myocardial infarction was defined as the presence of at least two of the three following conditions: (1) An appropriate clinical situation suggestive of an MI (e.g., abnormal history, physical examination, or new electrocardiogram changes); (2) troponin T or I ≥2 × upper limit of normal (ULN); (3) New, significant (≥0.04 seconds) Q waves in ≥2 contiguous leads.^3^

-Acute stroke was defined as a new, focal neurologic deficit of sudden onset, lasting at least 24 hours, not due to a readily identifiable nonvascular cause (i.e., brain tumor, trauma).^4^

**Pulmonary embolism early risk stratification**

Pulmonary embolism was risk-stratified according to the European Society of Cardiology (ESC) guidelines as low, intermediate-low, intermediate-high, and high risk:^5^

-Patients with cardiac arrest, obstructive shock or persistent hypotension were stratified as **high-risk.**

-Hemodynamically stable patients with simplified Pulmonary Embolism Severity Index (sPESI) ≥1, RV dysfunction, and positive troponin were stratified as **intermediate-high risk.**

-Hemodynamically stable patients with sPESI ≥1 and/or RV dysfunction were stratified as **intermediate-low risk**.

The remaining hemodynamically stable patients with sPESI = 0, normal RV function and negative troponin were stratified as **low risk**.

**Right ventricle (RV) dysfunction**

*On CT-scan:*

-Increased end-diastolic RV/left ventricle diameter ≥1.0

*On trans-thoracic echocardiography:*

-Enlarged right ventricle, parasternal long axis view

-Dilated RV with basal RV/LV ratio >1.0, and McConnell sign (arrow), four chamber view

- Flattened intraventricle septum parasternal short axis view

- 60/60 sign: coexistence of acceleration time of pulmonary ejection <60 ms and midsystolic “notch” with mildy elevated (<60 mmHg) peak systolic gradient at the tricuspic valve

- Right heart mobile thrombus detected in right heart cavities

- Decreased tricuspid annular plane systolic excursion (TAPSE) measured with M-Mode
(<16 mm)

**Table S1: The simplified Pulmonary Embolism Severity Index (sPESI):^6^**

| **Variable** | **Points assigned** |
| --- | --- |
| Age >80 years | **+1** |
| History of cancer | **+1** |
| History of chronic cardiopulmonary disease | **+1** |
| Pulse rate ≥ 110 beats/min | **+1** |
| Systolic blood pressure <100 mmHg | **+1** |
| Arterial oxyhemoglobin saturation (SaO2) < 90% | **+1** |

A total point score for a given patient is obtained by summing the points. The score corresponds with following risk classes; 0, low risk (mortality at 30 days 1%); ≥ 1, high risk (mortality at 30 days 10%).

**Table S2: List of candidate covariates for the multivariable models performed in the present analysis after acute pulmonary embolism**

| **Covariates** | **Missing data (%)** |
| --- | --- |
| Age, year | 0 |
| Age (per quartile) |  |
| Female sex (%) | 0 |
| **Comorbidities (%)** |  |
| Current smoker | 0 |
| Hypertension | 0 |
| Diabetes mellitus | 0 |
| Dyslipidemia | 0 |
| Chronic pulmonary disease | 0 |
| Coronary artery disease | 0 |
| Cancer | 0 |
| Prior stroke | 0 |
| Prior bleeding | 1.2 |
| Neurocognitive disorders | 0.3 |
| Liver disease | 0 |
| Excessive Fall Risk | 0.4 |
| Ethanol abuse | 0 |
| Recent surgery | 0 |
| Recent hospitalization | 0 |
| Thrombophilia | 0 |
| Pregnancy | 0 |
| Post-partum period | 0 |
| Immobility due to sitting (e.g. prolonged car or air travel) | 0 |
| Bed rest > 3 days | 0 |
| Varicose veins | 0 |
| Associated DVT | 0 |
| Clinical parameters at admission |  |
| Syncope | 0.5 |
| Heart rate, b.p.m | 0 |
| Heart rate (per quartile) |  |
| Systolic blood pressure, mmHg | 0 |
| Systolic blood pressure (per quartile) |  |
| Oxygen saturation, % | 0.6 |
| Oxygen saturation (per quartile) |  |
| **Biological data at inclusion** |  |
| Positive troponin | **0.5** |
| Hemoglobin, g/dL | 0.6 |
| Hemoglobin (per quartile) |  |
| Platelet count, x 10^3^/microliter | 0.7 |
| Platelet count (per quartile) |  |
| eGFR, mmol/L | 0 |
| eGFR_MDRD4_ (per quartile) |  |
| **Echocardiographic data at admission** | 1.1 |
| **Right Ventricle dysfunction** |  |

VTE, venous thromboembolic; DVT, deep vein thrombosis; eGFR, estimated glomerular function; MDRD4, the four variables the Modification of Diet in Renal Disease equation;

**Table S3: Baseline characteristics of patients admitted to the participating centers with a diagnosis of confirmed PE during the study period (n=2,976), according to the BMI-defined underweight status or non-underweight status.**

|  | **Underweight^a^** | |
| --- | --- | --- |
| **Variables** | **No**  **(n=2,390)** | **Yes**  **(n=586)** |
| Age, years | 66.9 ± 16.8 | 68.8 ± 18.7 |
| Male (%) | 1188 (49.7) | 266 (45.4) |
| BMI (kg/m^2^) | 27.6 ± 5.9 | 17.2 ± 1.3 |
| **Co-morbidities (%)** |  |  |
| Pulmonary disease/HF | 189 (7.9) | 63 (10.7) |
| Prior stroke | 118 (4.9) | 42 (7.2) |
| Active cancer^b^ | 452 (18.9) | 132 (22.5) |
| Prior VTE | 557 (23.3) | 112 (19.1) |
| Prior bleeding | 11 (2.4) | 4 (2.2) |
| Transient or reversible factors of VTE | 576 (24.1) | 135 (23.0) |
| No identifiable risk factor (unprovoked PE) | 1814 (75.9) | 1309 (76.8) |
| Associated DVT | 977 (40.9) | 193 (32.9) |
| **Clinical characteristics** |  |  |
| Syncope at presentation | 161 (6.7) | 59 (10.1) |
| HR at admission (bpm) | 89.7 ± 18.4 | 92.3 ± 21.4 |
| SBP at admission (mmHg) | 138.6 ± 23.4 | 134.2 ± 24.6 |
| SaO^2^ (%) | 93.4 ± 58 | 93.3 ± 5.4 |
| **Biological data** |  |  |
| Positive troponin | 929 (38.9) | 199 (34.0) |
| Hemoglobin at admission | 13.3 ± 2.0 | 12.9 ± 2.2 |
| eGFR at admission | 82.0 ± 33.3 | 84.3 ± 34.3 |
| **Echo data** |  |  |
| RV dysfunction^c^ | 964 (40.3) | 159 (27.1) |
| **sPESI (points, Q1-Q3)** | 2 (1-3) | 2 (1-3) |
| **ESC-defined risk stratification of index PE (%)** |  |  |
| Low risk | 356 (14.9) | 60 (14.4) |
| Intermediate-low risk | 1356 (56.7) | 383 (65.4) |
| Intermediate-high risk | 611 (25.6) | 98 (16.7) |
| High-risk | 67 (2.8) | 45 (7.7) |

BMI: body mass index; VTE: venous thromboembolic; DVT: deep vein thrombosis; PE: pulmonary embolism; HR: heart rate; SBP: systolic blood pressure; SaO^2^, arterial oxyhemoglobin saturation; RV: right ventricle; sPESI, simplified Pulmonary Embolism Severity Index; ESC, The European Society of Cardiology

^a^ Body Mass Index <18.5 kg/m^2^; ^b^ Cancer is considered active when at least one of the following three conditions is met: 1) The patient has received a potentially non-curative treatment of his cancer (case in particular of so-called palliative chemotherapy); 2) the evolution shows recurrence or progression of the cancer under treatment and 3) the cancer treatment is ongoing; ^c^ RV dysfunction was defined by at least one of the following parameter: a right ventricle/left ventricle diameter ratio > 1.0 on echocardiography or CT-scan, flattened intraventricular septum on echography, peak systolic gradient at the tricuspid valve > 30 mmHg, or a tricuspid annular plane systolic excursion (TAPSE) < 16 mm;

**Table S4: In-hospital therapies of 2,390 patients with acute pulmonary embolism according to their body mass index status (obesity vs no obesity).**

|  | **Obesity^a^**  **(n = 686)** | **No obesity**  **(n = 1,704)** |
| --- | --- | --- |
| **Clinical characteristics** |  |  |
| **Anticoagulant at admission (%)** |  |  |
| Unfractionated heparin | 128 (18.7) | 253 (14.8) |
| LMWH | 353 (51.5) | 1,014 (59.5) |
| DOAC | 205 (29.9) | 437 (25.6) |
| VKA | - | - |
| **Advanced therapy (%)** |  |  |
| Advanced reperfusion therapy | 54 (7.9) | 64 (3.8) |
| Systemic thrombolysis | 52 (7.6) | 57 (3.3) |
| Surgical embolectomy | 4 (0.6) | 8 (0.5) |
| ECMO | 5 (0.7) | 9 (0.5) |
| IVC filter implantation | 1 (0.1) | 10 (0.6) |
| **Anticoagulant at discharge (%)** |  |  |
| None | 3 (0.4) | 9 (0.5) |
| LMWH | 137 (20.0) | 445 (26.1) |
| VKA | 89 (13.0) | 224 (13.1) |
| DOAC | 445 (64.9) | 1,026 (60.2) |
| Appropriate dosing of DOAC^b^ | 425 (98.4) | 961 (96.6) |
| **Adjusted hospital length of stay^c^** | 5.9 ± 6.3 | 6.0 ± 6.5 |

LMWH, low molecular weight heparin; DOAC, direct oral anticoagulant; VKA, vitamin K antagonist; ECMO, extra-corporeal membrane oxygenation; IVC, inferior vena cava

^a^ Body mass index (BMI) ≥ 30 kg/m2; ^b^ 20mg once daily for rivaroxaban or 5mg twice daily for apixaban (<https://www.fda.gov>); ^c^ Adjusted for covariates identified by multivariate analyses (i.e. age [per quartile], diabetes mellitus, prior coronary artery disease, prior stroke, active cancer, recent hospitalization, systolic blood pressure at admission [per quartile], oxygen saturation at admission [per quartile], right ventricle dysfunction, positive troponin).

**Table S5:** **Observed rates and incidence rates of clinical outcomes with associated unadjusted and adjusted risk estimates between obese and non-obese patients after acute pulmonary embolism (n=2,390).**

|  | **Obesity**^a^  **(n = 686)** | **No obesity**  **(n = 1,704)** |
| --- | --- | --- |
| **Outcomes at 30 days** |  |  |
| **Death from any cause** |  |  |
| Number of events | 22 (3.2%; 95% CI ,2.0-4.8) | 100 (5.9%; 95% CI, 4.8-7.1) |
| Incidence rates, per patient-years | 0.38 (95% CI,0.35-0.42) | 1.34 (95% CI,1.22-1.5) |
| Unadjusted risk estimates | 0.53 (95% CI, 0.33-0.85) | |
| Adjusted risk estimates | 0.54 (95% CI,0.32-0.92) | |
| **Major bleeding** |  |  |
| Number of events | 19 (2.8%; 95% CI, 1.7-4.3) | 35 (2.0%; 95% CI, 1.4-2.8) |
| Incidence rates, per patient-years | 0.33 (95% CI, 0.05-1.11) | 0.24 (095% CI, 0.07-0.61) |
| Unadjusted risk estimates | 1.35 (95% CI, 0.77-2.39) | |
| Adjusted risk estimates | 1.36 (95% CI, 0.74-2.49) | |
| **Clinically relevant non-major bleeding** |  |  |
| Number of events | 8 (1.1%; 95% CI, 6.1-10.3) | 28 (1.6%; 95% CI, 1.1-2.3) |
| Incidence rates, per patient-years | 0.14 (95% CI, 0.0-0.80) | 0.19 (95% CI, 0.04-0.53) |
| Unadjusted risk estimates | 0.70 (95% CI, 0.32-1.55) | |
| Adjusted risk estimates | 0.63 (0.28-1.42) | |
| **Outcomes à 6 months** |  |  |
| **Death from any cause** |  |  |
| Number of events | 56 (8.2%; 95% CI, .3-10.5) | 277 (16.3%; 95% CI, 14.6-18.1) |
| Incidence rates, per patient-years | 0.16 (95% CI, 0.01-0.83) | 0.32 (95% CI, 0.11-0.72) |
| Unadjusted risk estimates | 0.51 (95% CI, 0.38-0.69) | |
| Adjusted risk estimates | 0.60 (95% CI, 0.44-0.82) | |
| **Major bleeding** |  |  |
| Number of events | 31 (4.5%; 95% CI, 3.1-6.3) | 70 (4.1%, 95% CI, 3.2-5.1) |
| Incidence rates, per patient-years | 0.09 (95% CI, 0.0-0.71) | 0.08 (95% CI, 0.0-0.36) |
| Unadjusted risk estimates | 1.04 (95%CI, 0.67-1.62) | |
| Adjusted risk estimates | 1.11 (95% CI, 0.71-1.75) | |
| **Clinically relevant non-major bleeding** | 25 (3.6%; 95%CI, 2.3-5.3) | 57 (3.3%; 95% CI, 2.5-4.3) |
| Number of events | 25 (3.6%; 95%CI, 2.3-5.3) | 57 (3.3%; 95% CI, 2.5-4.3) |
| Incidence rates, per patient-years | 0.07 (95%CI, 0.0-0.67) | 0.06 (95% CI, 0.0-0.33) |
| Unadjusted risk estimates | 1.40 (95% CI, 0.641.69) | |
| Adjusted risk estimates | 1.02 (95% CI,0.62-1.66) | |
| **Recurrent VTE** |  |  |
| Number of events | 7 (1.0%; 95% CI, 0.4-2.2) | 30 (1.8%; 95% CI, 1.2-2.5) |
| Incidence rates, per patient-years | 0.02 (95% CI, 0.0-0.58) | 0.03 (95% CI, 0.0-0.28) |
| Unadjusted risk estimates | 0.61 (95% CI, 0.27-1.41) | |
| Adjusted risk estimates | 0.57 (95%CI, 0.24-1.30) | |
| **Myocardial infarction** |  |  |
| Number of events | 5 (0.7%; 95% CI, 0.2-1.6) | 9 (0.5%; 95% CI, 0.2-1.0) |
| Incidence rates, per patient-years | 0.01 (95% CI, 0.0-0.12) | 0.01 (95% CI, 0.0-0.12) |
| Unadjusted risk estimates | 1.38 (95% CI, 0.46-4.11) | |
| Adjusted risk estimates | 1.66 (95% CI, 0.54-5.07) | |
| **Stroke** |  |  |
| Number of events | 1 (0.1%; 95% CI, 0.0-0.7) | 16 (0.9%; 95% CI, 0.5-1.5) |
| Incidence rates, per patient-years | 0.002 (95% CI, 0.0-0.0023) | 0.02 (95% CI, 0.0-0.26) |
| Unadjusted risk estimates | 0.15 (95% CI, 0.02-1.17) | |
| Adjusted risk estimates | 0.15 (95% CI, 0.02-1.18) | |

VTE, venous thromboembolism.

^a^ Body mass index (BMI) ≥ 30 kg/m^2^

Risk estimates with obesity as reference

**Table S6: Observed causes of death between obese and non-obese patients after acute pulmonary embolism (n=2,390).**

| **Cause of death** | **Obesity**^a^  **(n = 686)** | **No obesity**  **(n = 1,704)** |
| --- | --- | --- |
| **At 30 days** |  |  |
| **VTE-related** | 11 (1.6%; 95% CI, 00.8-2.8)) | 23 (1.3%; 95% CI, 0.8-2.0) |
| **Cancer-related** | 4 (0.6%; 95% CI, 0.2-1.5) | 44 (2.6%; 95% CI, 1.9-3.5) |
| **Bleeding-related** | 3 (0.4%; 95% CI, 0.07-1.2) | 2 (0.1%; 95% CI, 0.01-0.4) |
| **Other** | 2 (0.3%; 0.04-1.1) | 23 (1.3%; 95% CI, 0.8-2.0- |
| **At 6 months** |  |  |
| **VTE-related** | 14 (2.0%; 95% CI, 1.1-3.3) | 31 (1.9%; 95% CI, 1.3-2.7) |
| **Cancer-related** | 23 (13.1%; 10.7-15.9) | 153 (9.0%; 7.7-10.5) |
| **Bleeding-related** | 4 (0.2%; 95% CI, 0.05-0.55) | 3 (0.4%; 95% CI, 0.07-1.1) |
| **Other** | 76 (4.5%; 95% CI, 3.6-5.6) | 11 (1.6%; 5% CI, 0.8-2.8) |

VTE, venous thromboembolism.

^a^ Body mass index (BMI) ≥ 30 kg/m^2^

**Table S7:** **Rates of major bleeding between obese and non-obese patients after acute pulmonary embolism (n=2,390).**

| **Criteria for major bleeding** | **Obesity^a^**  **(n = 686)** | **No obesity**  **(n = 1,704)** |
| --- | --- | --- |
| **At 30 days** |  |  |
| Fatal bleeding | 3 (0.4%; 95% CI, 0.07-1.2) | 2 (0.1%; 95% CI, 0.01-0.4) |
| Fall in hemoglobin level of 20 g/L | 2 (0.3%; 95% CI, 0.01-0.9) | 12 (0.7%; 95% CI, 0.4-1.2) |
| Transfusion of ≥2 units of whole blood or red blood cells | 10 (1.5%; 95% CI, 0.7-2.7) | 14 (0.8%; 95% CI, 0.4-1.3) |
| Intra-cranial bleeding | 5 (0.7%; 95% CI, 0.2-1.6) | 8 (0.5% ; 95% CI, 0.2-1.0) |
| Symptomatic bleeding in a critical area or organ^b^ | 6 (0.9%; 95% CI, 0.3-1.9) | 14 (0.8%; 95% CI, 0.4-1.3) |
| **At 6 months** |  |  |
| Fatal bleeding | 4 (0.2%; 95% CI, 0.05-0.55) | 3 (0.4%; 95% CI, 0.07-1.1) |
| Fall in hemoglobin level of 20 g/L | 10 (1.5%; 95% CI, 0.7-2.7) | 31 (1.8%; 95% CI, 1.2-2.5) |
| Transfusion of ≥2 units of whole blood or red blood cells | 11 (1.6%; 95% CI 0.8-2.8) | 21 (1.2%; 95%CI, 0.7-1.8) |
| Intra-cranial bleeding | 6 (0.9%; 95% CI, 0.3-1.9) | 15 (0.9%; 95% CI, 0.5-1.5) |
| Symptomatic bleeding in a critical area or organ^b^ | 7 (1.0%; 95% CI, 0.4-2.1) | 27 (1.6%; 95% CI, 1.1-2.3) |

^a^ Body mass index (BMI) ≥ 30 kg/m^2^; ^b^ Intracranial, intraspinal, intraocular, retroperitoneal, intra‐articular or pericardial, or intramuscular with compartment syndrome

**Table S8: Univariate and multivariate predictors of the primary and secondary outcomes at 30 days and 6 months after acute pulmonary embolism (n = 2,390).**

| **Variable** | **HR (95% CI)** |
| --- | --- |
| **Primary outcomes** |  |
| **All-cause mortality at 30 days** |  |
| RCS transformed BMI | - |
| Who-defined obesity^a^ | 0.53 (0.33-0.85) |
| Age, per quartile | 1.21 (1.02-1.40) |
| Active cancer^b^ | 5.34 (3.68-7.75) |
| Prior CAD | 2.74 (1.14-6.58) |
| Syncope at PE presentation | 2.67 (1.57-4.53) |
| Positive troponin at admission | 3.43 (2.33-5.06) |
| RV dysfunction at admission^c^ | 1.46 (1.01-2.10) |
| SBP at admission, per quartile | 1.52 (1.28-1.81) |
| Hemoglobin at admission, per quartile | 1.56 (1.31-1.85) |
| In-hospital systemic thrombolysis | 3.24 (1.81-5.78) |
| **All-cause mortality at 6 months** |  |
| RCS transformed BMI | - |
| Age, per quartile | 1.25 (1.13-1.3) |
| COPD | 1.83 (1.32-2.54) |
| Active cancer^b^ | 7.34 (5.86-9.20) |
| Diabetes mellitus | 1.48 (1.10-1.99) |
| Prior stroke | 1.65 (1.08-2.53) |
| No identifiable risk factor (unprovoked PE) | 1.68 (1.25-2.25) |
| Positive troponin | 1.67 (1.34-2.09) |
| Heat rate at admission, per quartile | 1.19 (1.08-1.31) |
| SBP at admission, per quartile | 1.36 (1.23-1.51) |
| Oxygen saturation, per quartile | 1.10 (1.00-1.21) |
| Hemoglobin at admission, per quartile | 1.76 (1.58-1.97) |
| In-hospital systemic thrombolysis | 1.62 (1.06-2.48) |
| **Secondary outcomes** |  |
| **Major bleeding at 30 days** |  |
| RCS transformed BMI | - |
| Who-defined obesity^a^ | 1.35 (0.77-2.39) |
| Age, per quartile | 1.34 (1.04-1.72) |
| Prior CAD | 5.44 (2.06-14.3) |
| Recent hospitalization^d^ | 5.03 (1.47-17.1) |
| Recent surgery^e^ | 2.16 (1.00-4.65) |
| Syncope at PE presentation | 2.97 (1.42-6.21) |
| RV dysfunction at admission^c^ | 2.02 (1.17-3.49) |
| Positive troponin at admission | 2.52 (1.45-4.39) |
| eGFR^f^ at admission, per quartile | 1.57 (1.20-2.04) |
| Hemoglobin at admission, per quartile | 1.90 (1.43-2.53) |
| Heat rate at admission, per quartile | 1.36 (1.06-1.74) |
| In-hospital systemic thrombolysis | 5.13 (2.51-10.5à |
| **CRNM bleeding at 30 days** |  |
| RCS transformed BMI | - |
| Who-defined obesity^a^ | 0.70 (0.32-1.55) |
| Positive troponin at admission | 2.50 (1.27-4.92) |
| In-hospital systemic thrombolysis | 3.49 (1.33-9.15) |
| **Outcomes at 6 months** |  |
| **All-cause mortality** |  |
| RCS transformed BMI | - |
| Age, per quartile | 1.25 (1.13-1.3) |
| COPD | 1.83 (1.32-2.54) |
| Active cancer^b^ | 7.34 (5.86-9.20) |
| Diabetes mellitus | 1.48 (1.10-1.99) |
| Prior stroke | 1.65 (1.08-2.53) |
| No identifiable risk factor (unprovoked PE) | 1.68 (1.25-2.25) |
| Positive troponin | 1.67 (1.34-2.09) |
| Heat rate at admission, per quartile | 1.19 (1.08-1.31) |
| SBP at admission, per quartile | 1.36 (1.23-1.51) |
| Oxygen saturation, per quartile | 1.10 (1.00-1.21) |
| Hemoglobin at admission, per quartile | 1.76 (1.58-1.97) |
| In-hospital systemic thrombolysis | 1.62 (1.06-2.48) |
| **Major bleeding at 6 months** |  |
| RCS transformed BMI | - |
| Who-defined obesity^a^ | 1.04 (0.67-1.62) |
| Age, per quartile | 1.26 (1.05-1.51) |
| Recent surgery^e^ | 1.97 (1.01-3.54) |
| Syncope at PE presentation | 2.56 (1.45-4.52) |
| Positive troponin at d-admission | 1.56 (1.04-2.33) |
| Heart rate at admission | 1.26 (1.05-1.52) |
| eGFR^f^ at admission, per quartile | 1.32 (1.10-1.59) |
| Hemoglobin at admission | 1.76 (1.44-2.17) |
| In-hospital systemic thrombolysis | 2.60 (1.35-5.01) |
| **CRNM bleeding at 6 months** |  |
| RCS transformed BMI | - |
| Who-defined obesity^a^ | 1.04 (0.64-1.69) |
| - | - |
| **Recurrent VTE** |  |
| RCS transformed BMI | - |
| Who-defined obesity^a^ | 0.53 (0.33-0.85) |
| Age, per quartile | 1.48 (1.08-2.04) |
| **Myocardial infarction at 6 months** |  |
| RCS transformed BMI | - |
| Who-defined obesity^a^ | 1.38 (0.46-4.11) |
| Age, per quartile | 2.05 (1.15-3.65) |
| eGFR, per quartile | 1.79 (1.04-3.07) |
| Hemoglobin at admission, per quartile | 1.69 (1.00-2.87) |
| **Stroke** |  |
| RCS transformed BMI | - |
| Who-defined obesity^a^ | 0.15 (0.02-1.17) |
| Prior stroke | 4.17 (1.20-14.5) |

RCS, restricted cubic spline; BMI, body mass index; CAD, coronary artery disease; SBP, systolic blood pressure; PE, pulmonary embolism; CNRM Clinically relevant non-major bleeding.

^a d^efined as body mass index (BMI) ≥ 30 kgm^2^; ^b^ Defined as a hemoglobin level < 12 g/dL; ^c^ RV dysfunction was defined by at least one of the following parameter: a right ventricle/left ventricle diameter ratio > 1.0 on echocardiography or CT-scan, flattened intraventricular septum on echography, peak systolic gradient at the tricuspid valve > 30 mmHg, or a tricuspid annular plane systolic excursion (TAPSE) < 16 mm; ^d^ > = 4 days for any non-surgical reason in the past 2 months^; d^ defined as Any surgical intervention in the past 2 months; ^f^ Estimated glomerular function calculated with the Modification of Diet in Renal

**Table S9: Overall model fit, discrimination, calibration indices, and risk reclassification indices when body mass index (transformed with restricted cubic spline function) is added or not to the European Society of Cardiology (ESC) model for the prediction of the 30-day all-cause death after acute pulmonary embolism in the study population and in the RIETE cohort for external validation.**

|  | **Study population (n =2,390)** | | **External validation (n =35,796)** | |
| --- | --- | --- | --- | --- |
|  | **ESC model with RCS-transformed BMI^a^** | | **ESC model with RCS-transformed BMI^a^** | |
|  | No | Yes | No | Yes |
| **Overall model fit** |  | | | |
| Bayes information criteria | 942.7 | 942.2 | 12144.8 | 12070.5 |
| Akaike information criteria | 931.2 | 924.8 | 12127.9 | 12045.1 |
| Nagelkerke’s R^2^ | 1.6% | 0.06% | 0.04% | 0.15% |
| **Discrimination** |  | | | |
| Harrell’s c index | 0.636 | 0.657^b^ | 0.654 | 0.673^c^ |
| **Calibration** |  | | | |
| P Hosmer-Lemeshow | 0.015 | 0.98 | <0.001 | 0.48 |
| **Risk reclassification between ESC model and ESC model with RCS-transformed BMI** | | | | |
| IDI | 3.4% (95% CI, 0.5-6.3 ; p = 0.02) | | 3.3% (95% CI, 0.4-2.6 ; p <0.001) | |
| Continuous NRI | 22.4% (95% CI, 8.3-36.6 ; p = 0.01) | | 23.1% (95% CI, 18.8-27.3 ; p <0.001) | |

RCS, restricted cubic splines; BMI, body mass index; OR, odds ratio; IDI: integrated discrimination improvement; NRI: net reclassification improvement; CI: confidence interval.

**^a^** defined by a Body Mass Index ≥ 30 kg/m^2^; ^b^Difference in Harrell’s c indices with p-value = 0.007; ^c^ Difference in Harrell’s c indices with p-value < 0.001.

**Table S10: Baseline characteristics of 35,166 patients with acute pulmonary embolism included in the RIETE population.**

| **Variables** | **Obesity**^a^  **(n=11,406)** | **No obesity**  **(n=23,760)** |
| --- | --- | --- |
| Age, years | 65.5 ± 15.7 | 66.4 ± 17.3 |
| Male (%) | 4,564 (40.0) | 12,389 (52.1) |
| BMI (kg/m^2^) | 34.7 ± 4.9 | 25.5 ± 2.7 |
| **Co-morbidities (%)** |  |  |
| Heart failure | 1,046 (9.2) | 2,000 (8.4) |
| Pulmonary disease | 1,645 (14.4) | 3,335 (14.1) |
| Prior stroke | 572 (5.0) | 1,381 (5.8) |
| Active cancer^b^ | 1,382 (12.1) | 4,282 (18.0) |
| Prior VTE | 1,779 (15.6) | 3,226 (13.6) |
| Prior bleeding | 180 (1;6) | 544 (2.3) |
| Transient or reversible factors of VTE | 4,203 (36.8) | 9,244 (38.9) |
| No identifiable risk factor (unprovoked PE) | 7,203 (63.1) | 14,516 (61.1) |
| Associated DVT | 4,415 (38.7) | 8,803 (37.0) |
| Family history of VTE | 291 (2.5) | 461 (1.9) |
| **Clinical characteristics** |  |  |
| Syncope at presentation | 1,570 (13.8) | 3,276 (13.8) |
| HR at admission (bpm) | 92.7 ± 19.9 | 91.9 ± 19.9 |
| SBP at admission (mmHg) | 132.6 ± 23.9 | 128.1 ± 23.4 |
| SaO^2^ (%) | 90.8 ± 6.9 | 91.2 ± 7.0 |
| **Biological data** |  |  |
| Positive troponin | 3,687 (32.3) | 7,990 (33.6) |
| Hemoglobin at admission | 13.2 ± 2.0 | 13.0 ± 2.2 |
| eGFR at admission | 75.9 ± 101.3 | 80.8 ± 129.1 |
| **Echo data** |  |  |
| RV dysfunction^c^ | 2,891 (25.3) | 5,446 (22.9) |
| **sPESI (points, Q1-Q3)** | 1 (0-1) | 1 (0-1) |
| **ESC-defined risk stratification of index PE (%)** |  |  |
| Low risk | 4,794 (42.0) | 9,185 (38.6) |
| Intermediate-low risk | 4,418 (38.7) | 9,543 (40.2) |
| Intermediate-high risk | 1,863 (16.3) | 4,268 (17.9) |
| High-risk | 331 (2.9) | 764 (3.2) |

BMI: body mass index; VTE: venous thromboembolic; DVT: deep vein thrombosis; PE: pulmonary embolism; HR: heart rate; SBP: systolic blood pressure; SaO^2^, arterial oxyhemoglobin saturation; RV: right ventricle; sPESI, simplified Pulmonary Embolism Severity Index; ESC, The European Society of Cardiology

^a^ Body mass index (BMI) ≥ 30 kg/m^2^; ^b^ Cancer is considered active when at least one of the following three conditions is met: 1) The patient has received a potentially non-curative treatment of his cancer (case in particular of so-called palliative chemotherapy); 2) the evolution shows recurrence or progression of the cancer under treatment and 3) the cancer treatment is ongoing; ^c^ RV dysfunction was defined by at least one of the following parameter: a right ventricle/left ventricle diameter ratio > 1.0 on echocardiography or CT-scan, flattened intraventricular septum on echography, peak systolic gradient at the tricuspid valve > 30 mmHg, or a tricuspid annular plane systolic excursion (TAPSE) < 16 mm.

**Figure S1: Study Flow-Chart**

**
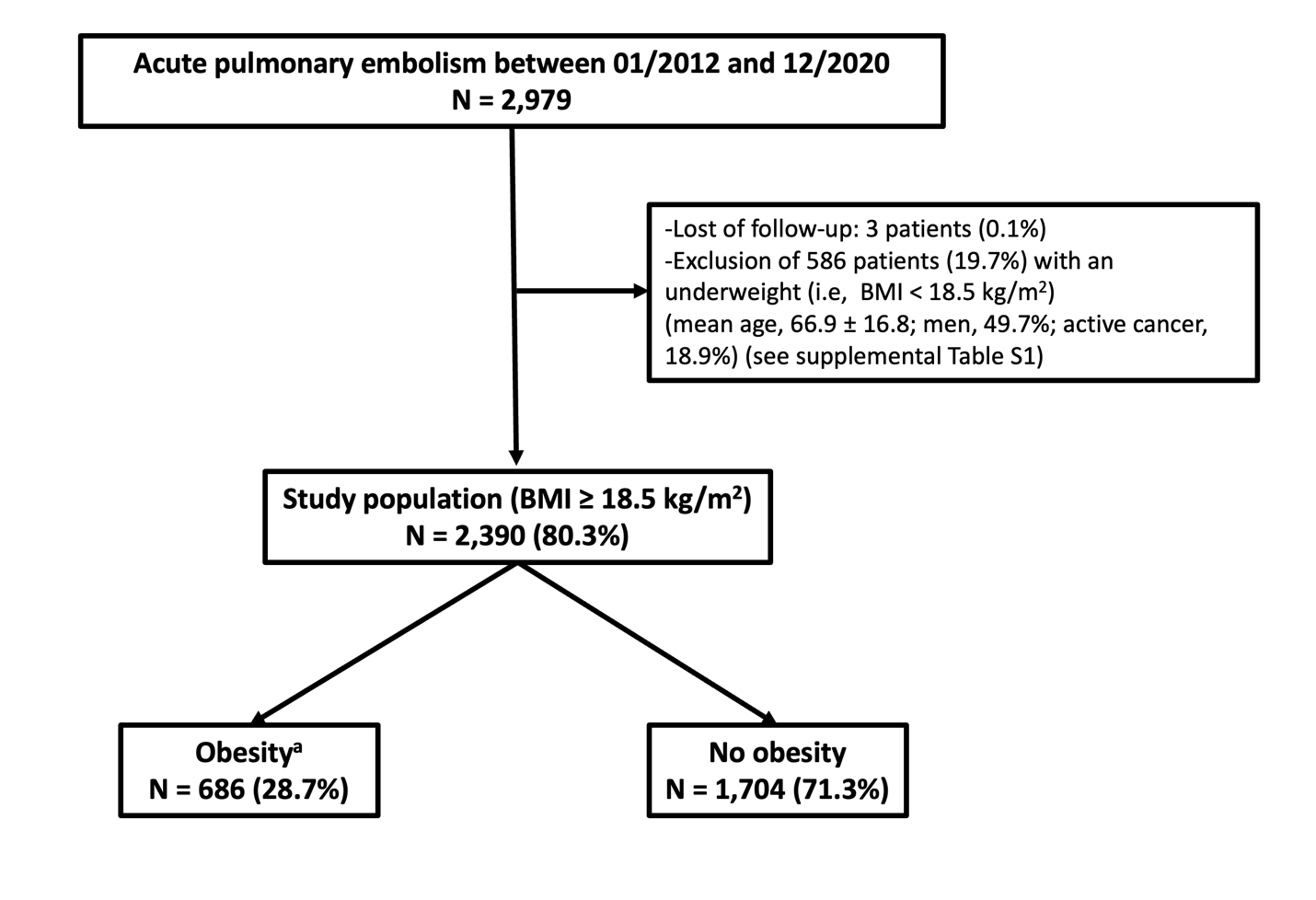
**

**Figure S2: Performances of the models evaluating the primary outcome (i.e., all-cause death) at 6 months (with assessment of the time-dependent Harrell’s C-indices for discrimination and the Hosmer-Lemeshow p-values for calibration)**

**
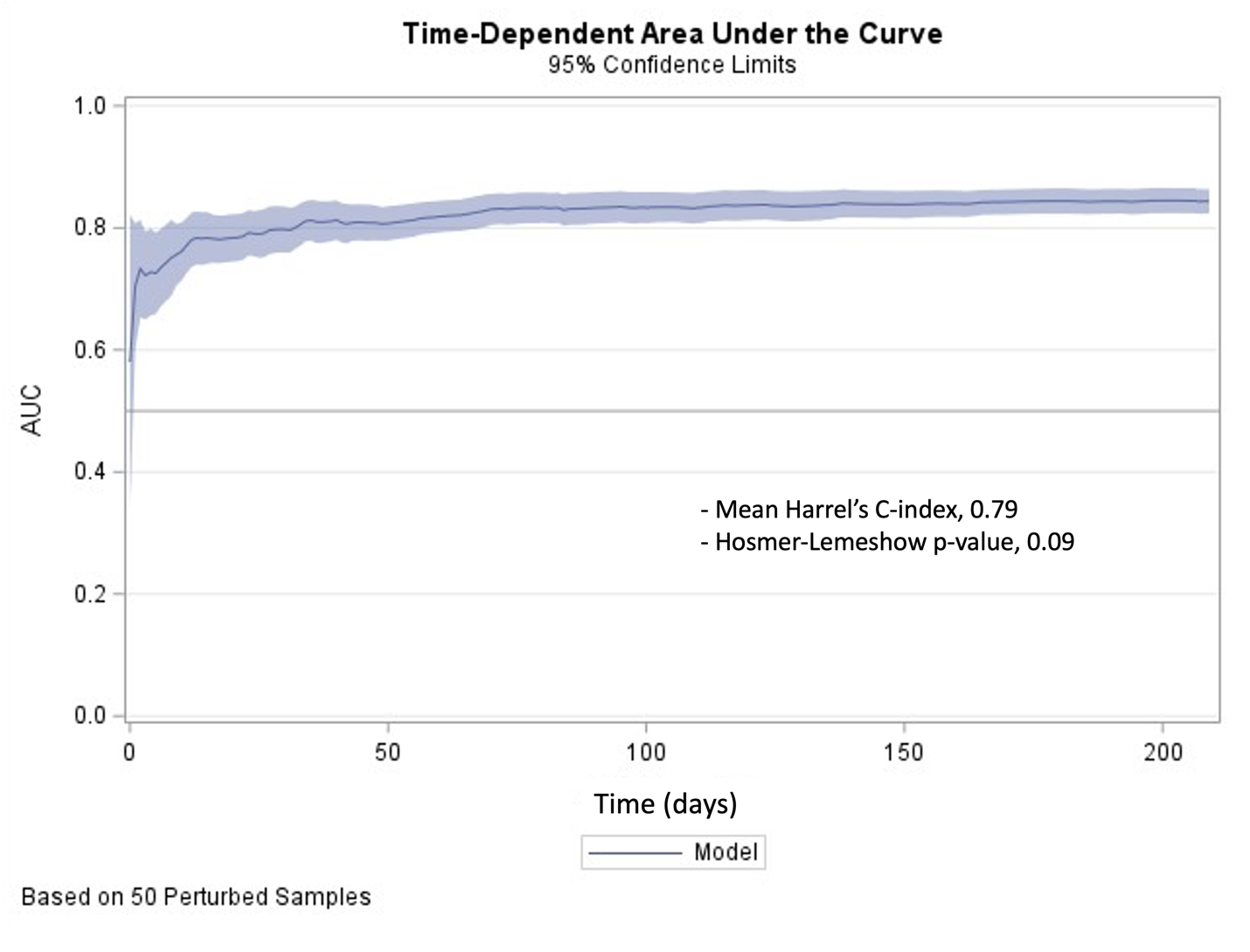
**

**Figure S3: Association between the body mass index function and primary outcomes, namely all-cause mortality at 30 days (A), and all-cause mortality at 6 months (B) using restricted cubic splines with 5 knots.**

Models adjusted with covariates included in the Supplemental Table S7.


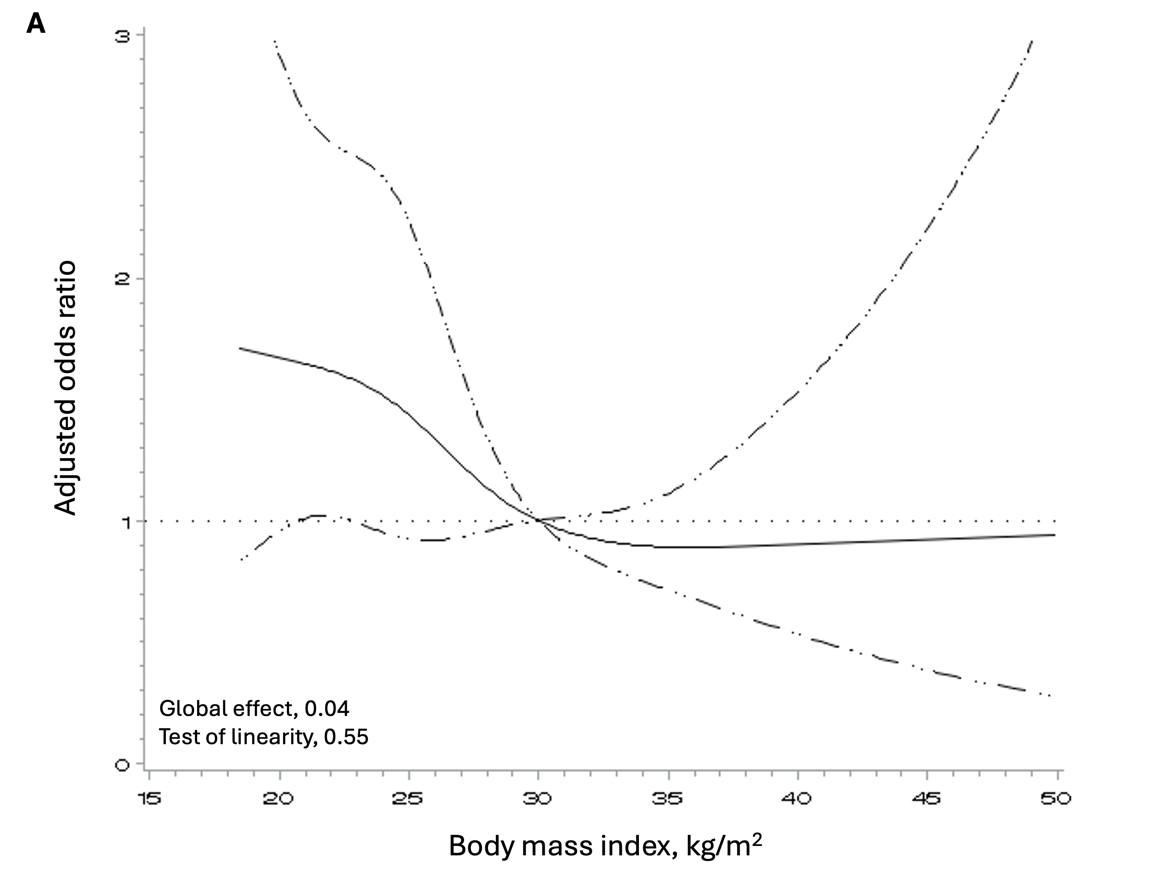


**
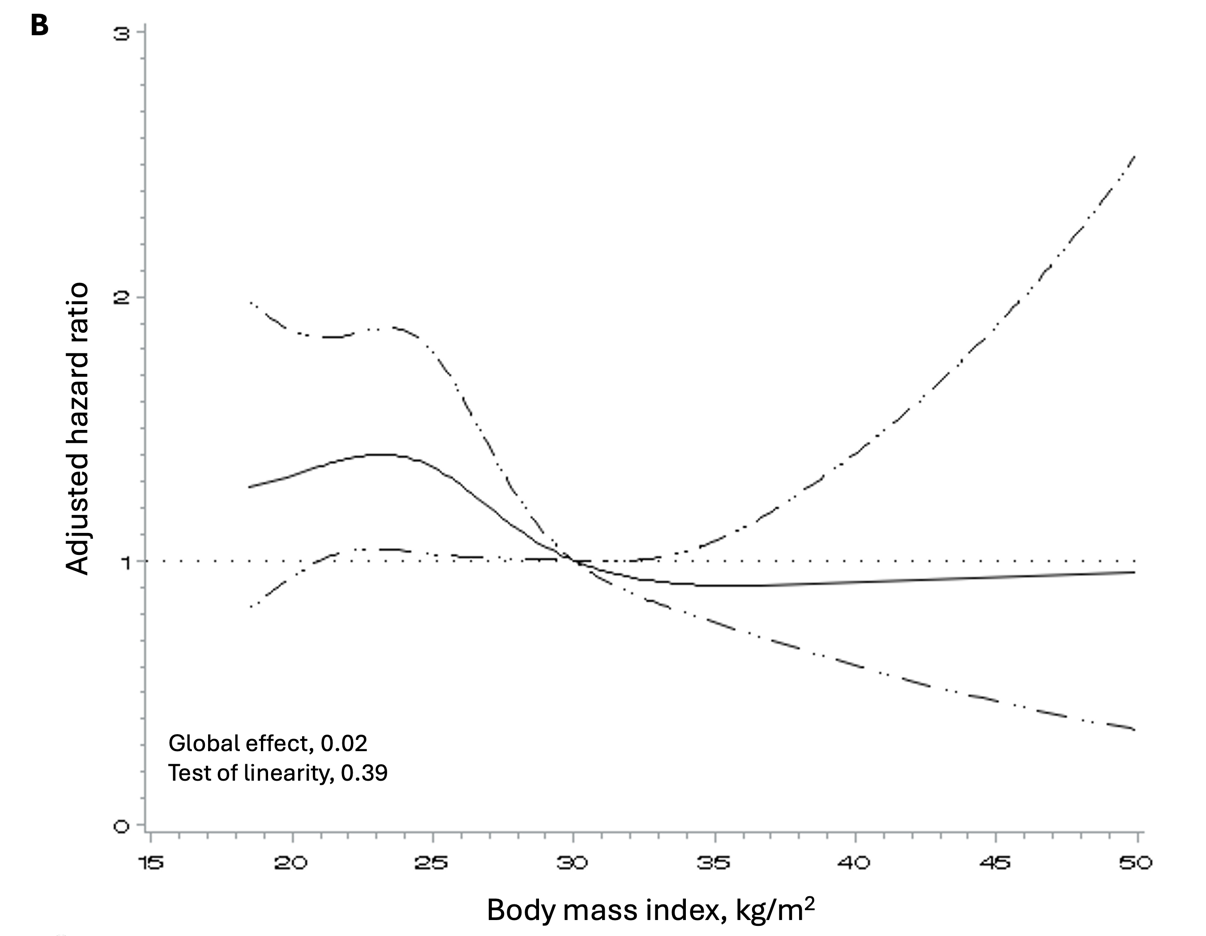
**

**Figure S4: Association between the body mass index function and secondary outcomes, namely major bleeding at 30 days (A), clinically relevant non-major bleeding at 30 days (B), major bleeding at 6 months (C), clinically relevant non-major bleeding at 6 months (D), recurrent venous thromboembolism at 6 months (E), myocardial infarction at 6 months (F), and stroke at 6 months (G) using restricted cubic splines with 5 knots.**

Models adjusted with covariates included in the Supplemental Table S7.


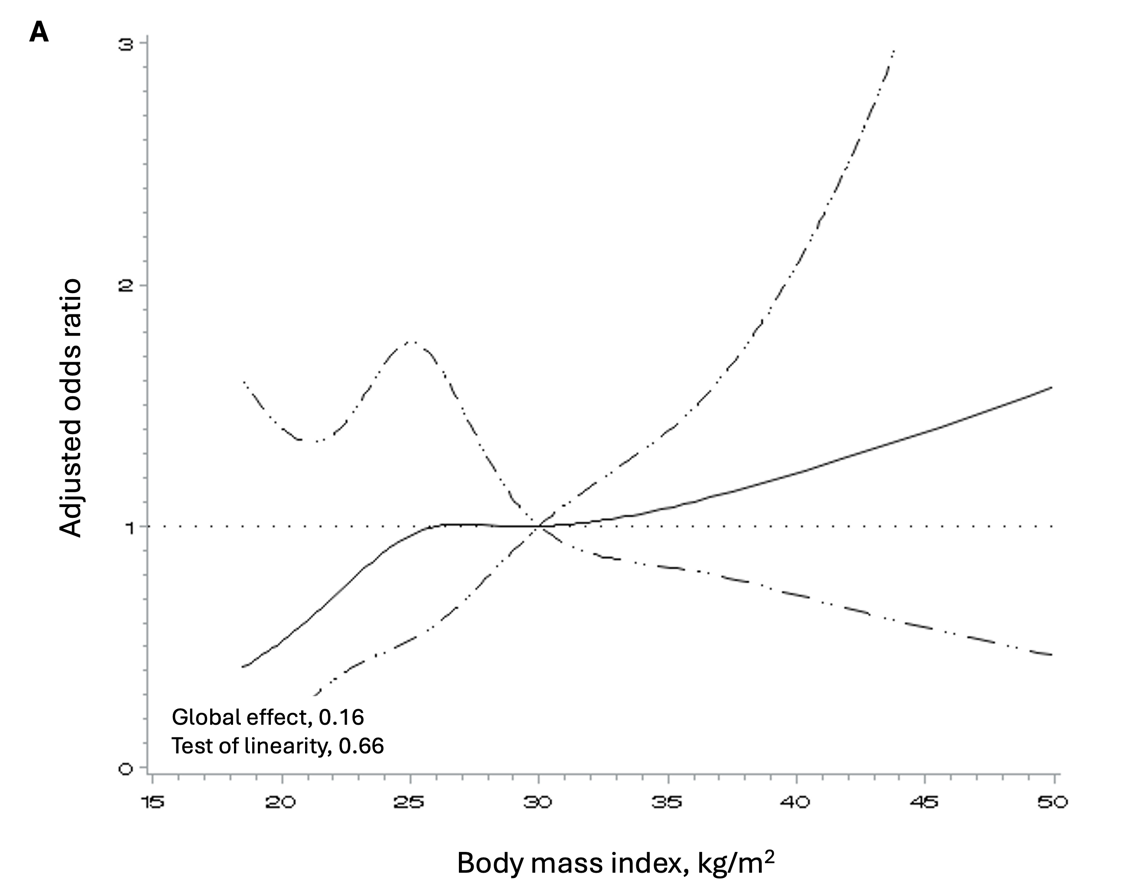


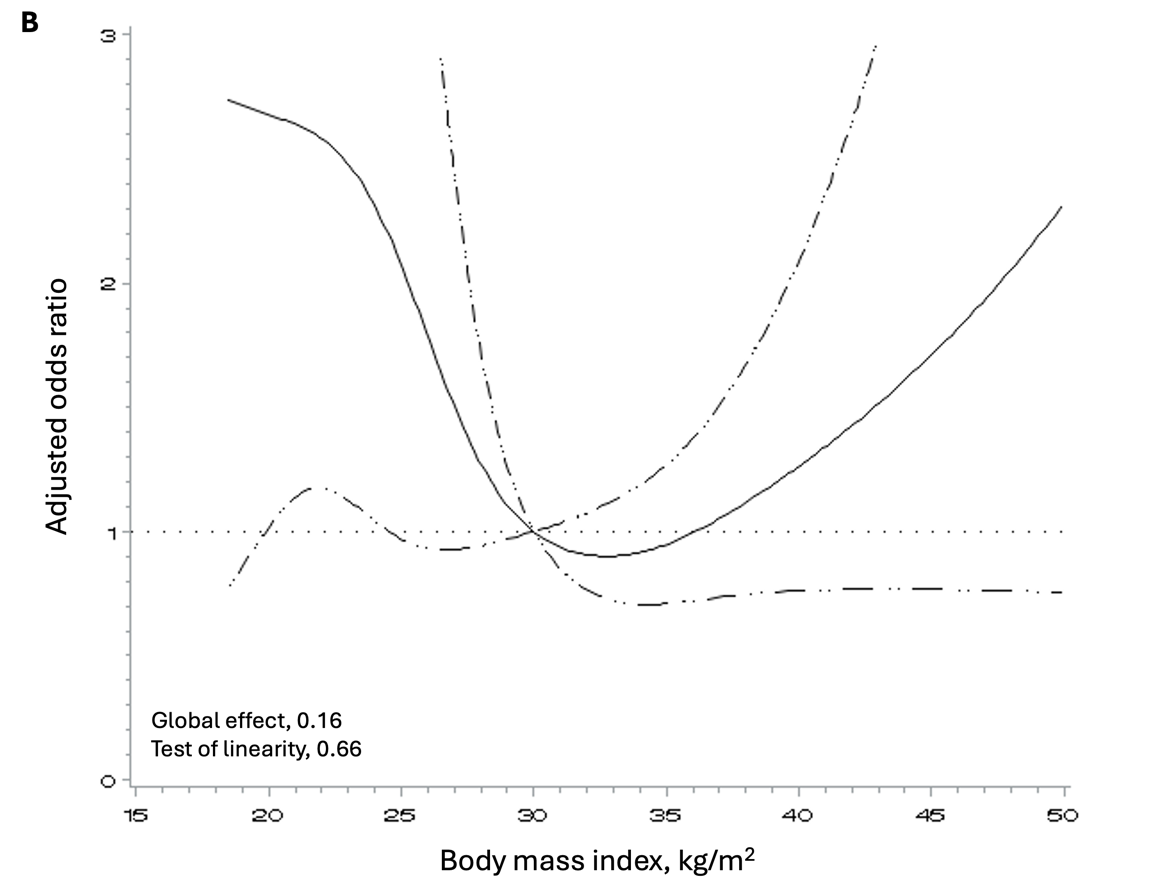


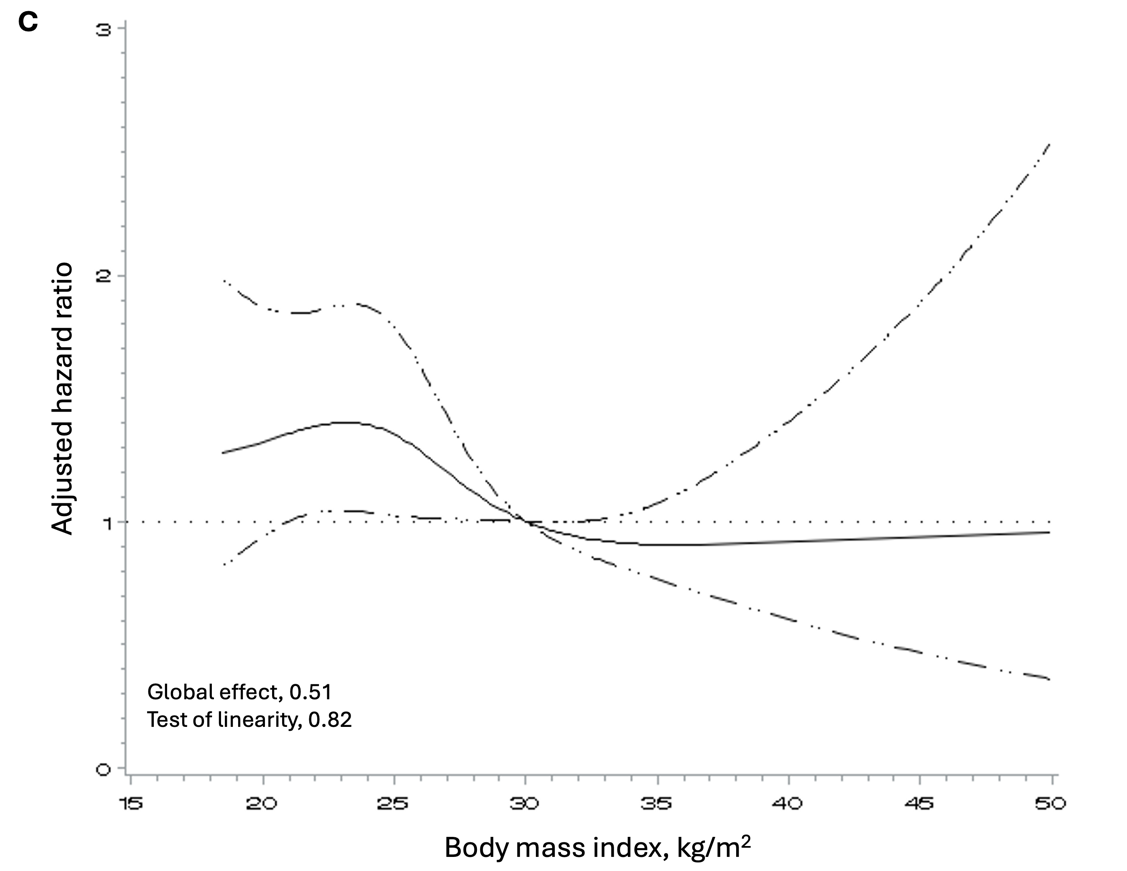


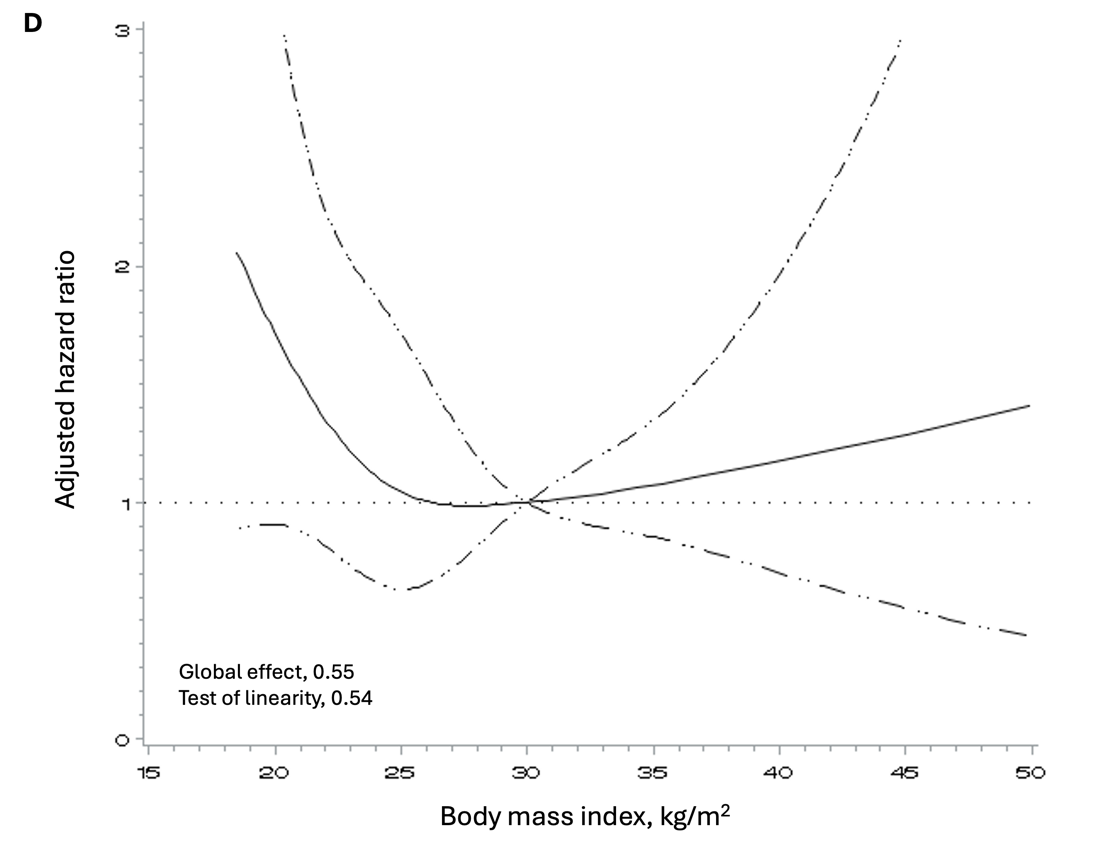


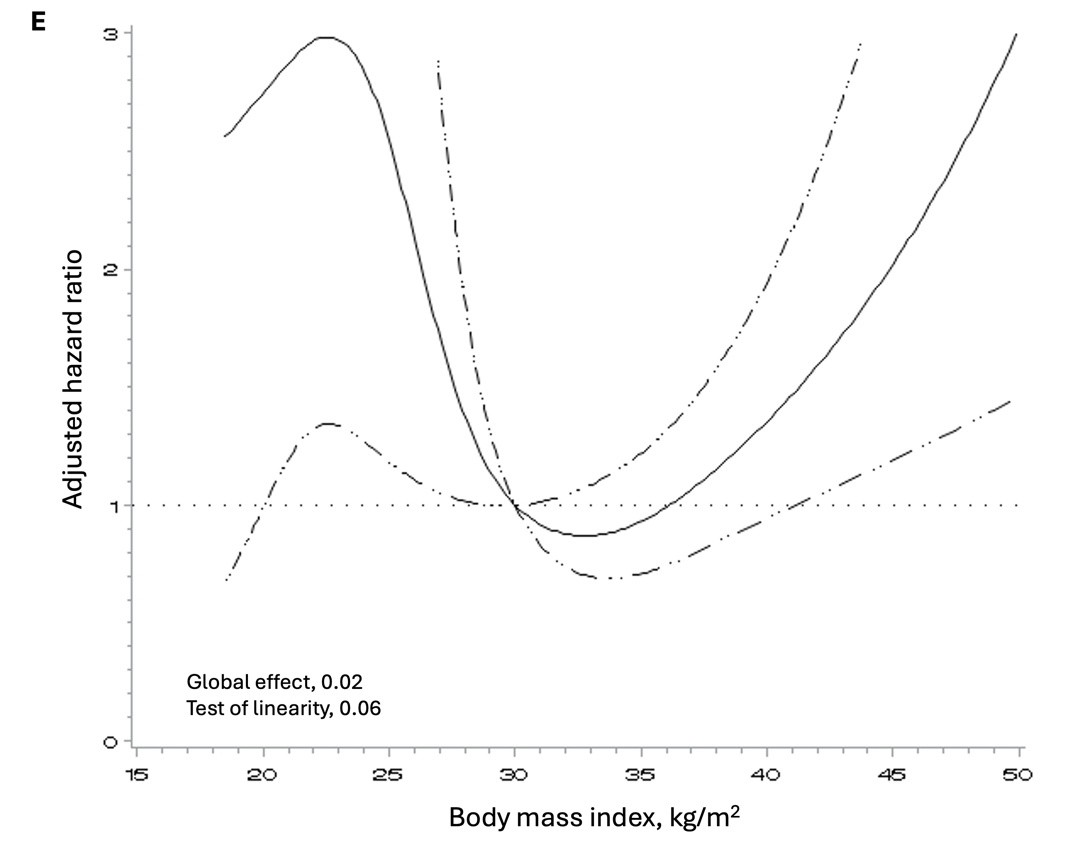


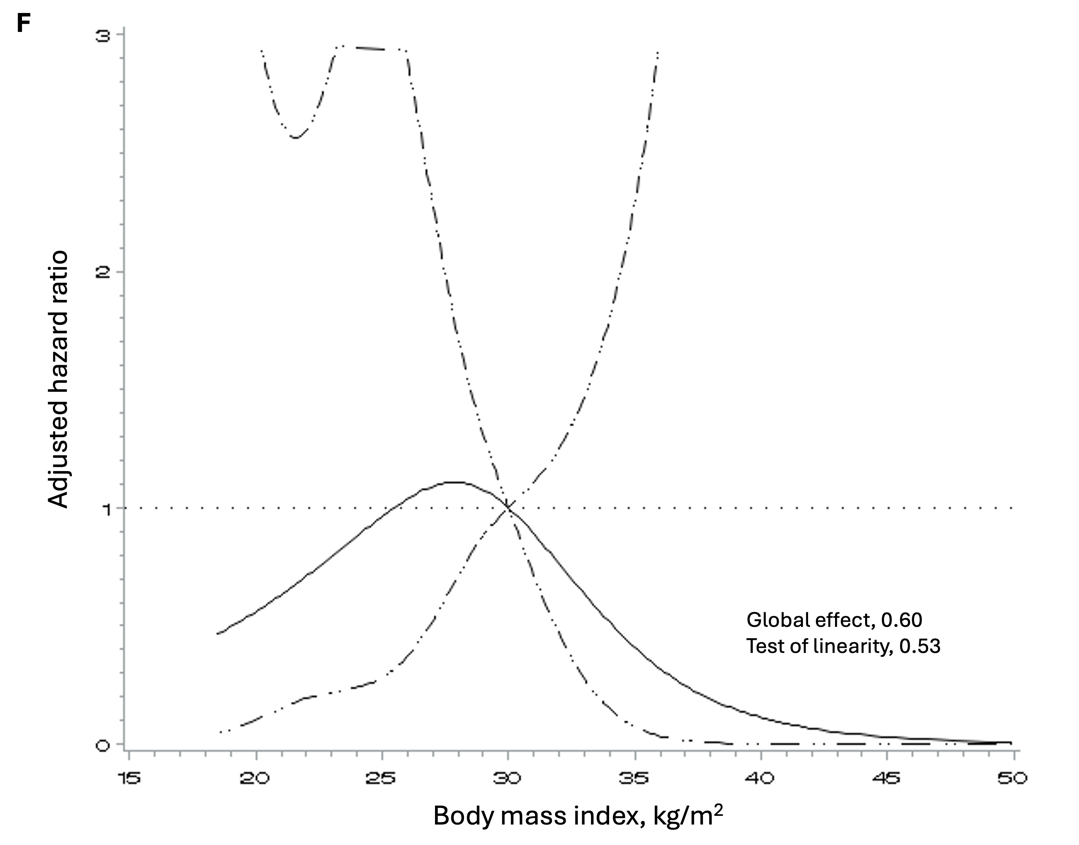
`


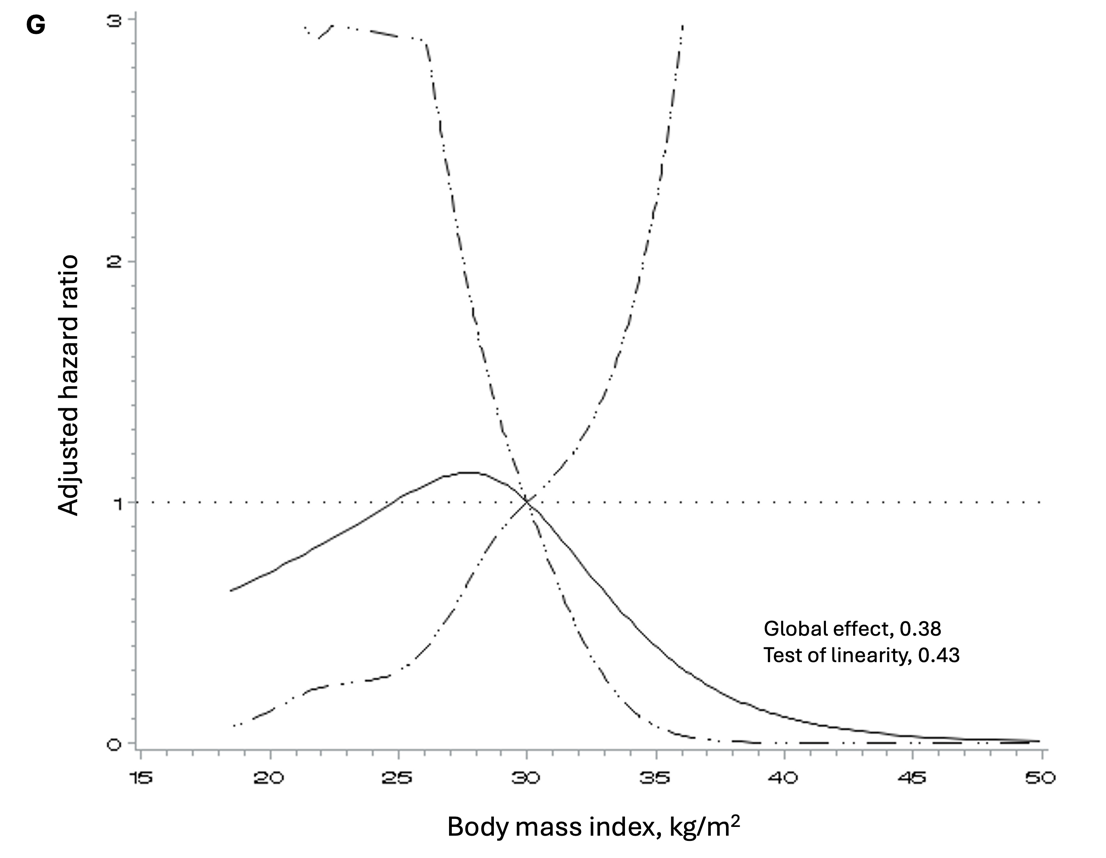


**Statistical approach with the use of inverse probability of treatment weighting (IPTW):**

**I**nverse probability of treatment weighting (IPTW) was used to adjust for confounding due to differences between groups, by assigning a weight of the mean of propensity scores (PS)/PS for the obese group and (1-means of PS)/(1 - PS) for the non-obese group, where PS is the probability that each individual will be assigned to early discharge. We computed the standardized mean difference to assess the balance of variables.^7^ Propensity scores were estimated by using a logistic regression model of the 17 covariates: age < 80 years, male sex, weight < 50 kg, active cancer, diabetes mellitus, prior coronary disease, prior stroke, prior VTE, unprovoked PE, syncope, renal dysfunction at admission, anemia at admission, heart rate > 110 bpm, systolic blood pressure < 100 mmHg, SOP2 <90%, RV dysfunction at admission, and positive troponin at admission. We analyzed the differences for the categorical and continuous variables between the 2 groups using a weighted chi-squared test and a weighted t-test, respectively.

After IPTW, we conducted weighted logistic and Cox proportional hazards regressions to identify the predictors of overall survival at 30 days and 6 months. We input the variables with P<0.1 resulting from the univariable regression into the multivariable regressions. We confirmed the linearity of continuous predictors on outcomes for regression and checked the proportional hazards assumption for the Cox regression using the correlation coefficient and the test of no correlation of the time to death and Schoenfeld residuals. We checked multicollinearity before the analysis of multivariable regression and considered variables with a variance influence factor > 4 to have multicollinearity.

**Figure S5: Absolute standardized mean difference in the unadjusted study population and after inverse probability of BMI-defined groups weighting.**

**
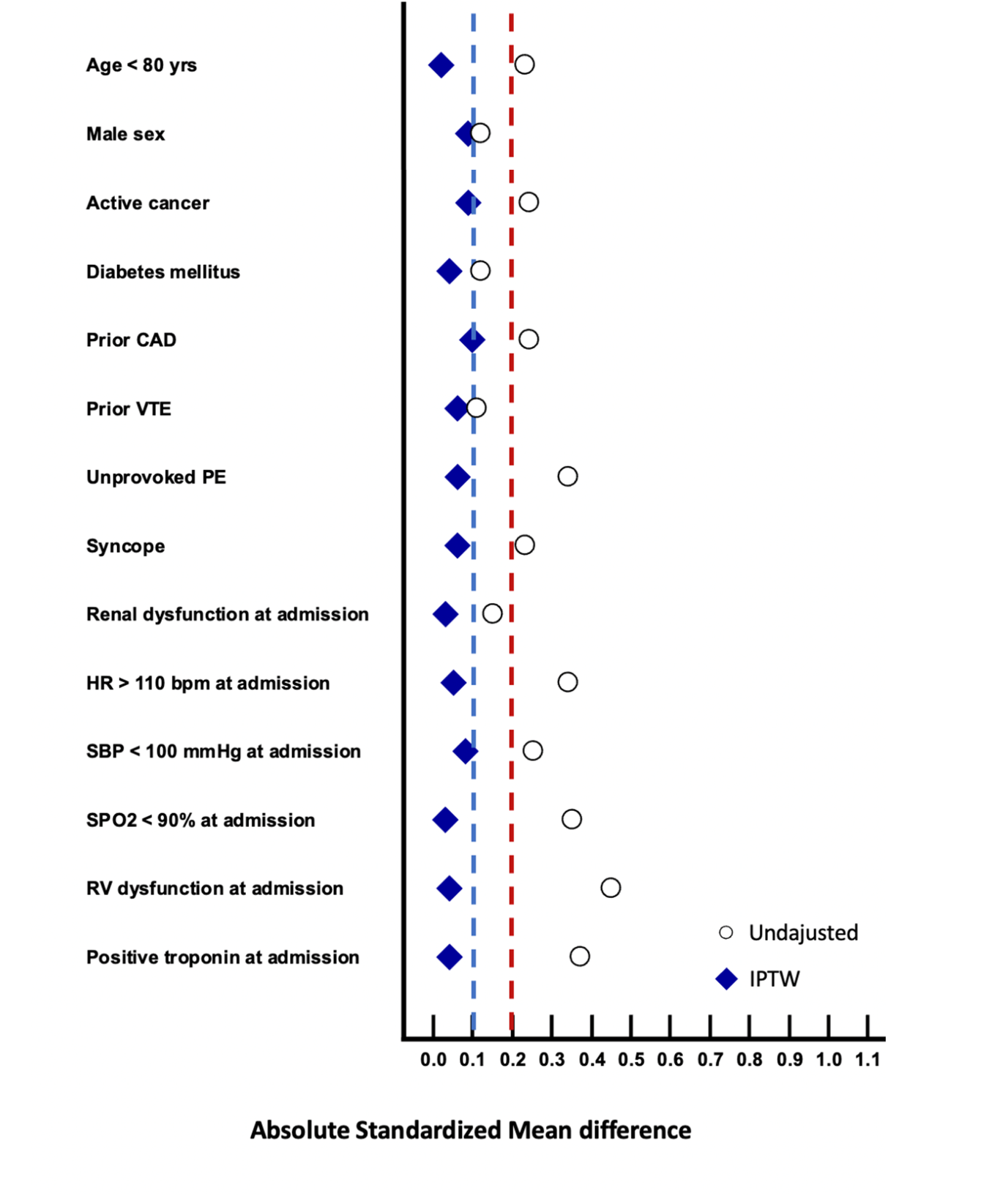
**

**Coordinator of the RIETE Registry:** Manuel Monreal.

**RIETE Steering Committee Members:** Paolo Prandoni, Benjamin Brenner and Dominique Farge-Bancel.

**RIETE National Coordinators:** Raquel Barba (Spain), Pierpaolo Di Micco (Italy), Laurent Bertoletti (France), Sebastian Schellong (Germany), Inna Tzoran (Israel), Abilio Reis (Portugal), Marijan Bosevski (R. Macedonia), Lucia Mazzolai (Switzerland), Radovan Malý (Czech Republic), Peter Verhamme (Belgium), Joseph A. Caprini (USA), Hanh My Bui (Vietnam).

**RIETE Registry Coordinating Center:**  S & H Medical Science Service.

**APPENDIX**

Members of the RIETE Group

**SPAIN**: Adarraga MD, Agudo P, Aibar J, Alberich-Conesa A, Alda-Lozano A, Alfonso J, Amado C, Angelina-García M, Arcelus JI, Ballaz A, Barba R, Barbagelata C, Barreiro B, Barrón M, Barrón-Andrés B, Bascuñana J, Beddar-Chaib F, Blanco-Molina A, Caballero JC, Cañas I, Casado I, Castellanos G, Criado J, De Juana-Izquierdo C, Del Toro J, Demelo-Rodríguez P, Díaz-Brasero AM, Díaz-Pedroche MC, Díaz-Peromingo JA, Dubois-Silva A, Escribano JC, Falgá C, Fernández-Aracil C, Fernández-Capitán C, Fernández-Jiménez B, Fernández-Reyes JL, Fidalgo MA, Francisco I, Gabara C, García-Bragado F, García-González C, García-Ortega A, Gavín-Sebastián O, Gil-Díaz A, Gómez-Cuervo C, González-Martínez J, González-Munera A, Grau E, Guirado L, Gutiérrez-Guisado J, Hernández-Blasco L, Hernández-Vidal MJ, Jara-Palomares L, Jiménez D, Jiménez R, Jou I, Joya MD, Lainez-Justo S, Lecumberri R, León-Ramírez JM, Llamas P, Lobo JL, López-De la Fuente M, López-Jiménez L, López-Miguel P, López-Núñez JJ, López-Ruiz A, López-Sáez JB, Lorente MA, Lorenzo A, Madridano O, Maestre A, Marchena PJ, Marcos M, Martín-Del Pozo M, Martín-Martos F, Martínez-Prado R, Maza JM, Mercado MI, Monreal M, Monzón L, Moragón-Ledesma S, Morales MV, Muñoz-Gamito G, Navas MS, Nieto JA, Núñez-Fernández MJ, Oblitas CM, Olid M, Ortiz M, Otálora S, Otero R, Pacheco-Gómez N, Pagán J, Parra-Caballero P, Pedrajas JM, Pérez-Ductor C, Pérez-Pinar M, Peris ML, Pesce ML, Porras JA, Puchades R, Rivas A, Rivera-Cívico F, Rivera-Gallego A, Rodríguez-Cobo A, Romero-Bruguera M, Ruiz-Artacho P, Ruiz-Giménez N, Salgueiro G, Sánchez-Serrano M, Sancho T, Sendín V, Sigüenza P, Soler S, Steinherr A, Suárez-Fernández S, Tirado R, Torrents-Vilar A, Torres MI, Trujillo-Santos J, Uresandi F, Valle R, Varona JF, Vicente-Navarro D, Villalobos A, Villares P, **AUSTRIA**: Ay C, Nopp S, Pabinger I, **BELGIUM**: Vanassche T, Verhamme P, Verstraete A, **BRAZIL**: Yoo HHB, **COLOMBIA**: Montenegro AC, Morales SN, Roa J, **CZECH REPUBLIC**: Hirmerova J, Malý R, **FRANCE**: Acassat S, Bertoletti L, Bura-Riviere A, Catella J, Chopard R, Couturaud F, Espitia O, Le Mao R, Leclerq B, Mahé I, Moustafa F, Plaisance L, Poenou G, Sarlon-Bartoli G, Suchon P, Versini E, **GERMANY**: Schellong S, **IRAN**: Rashidi F, Sadeghipour P, **ISRAEL**: Brenner B, Dally N, Kennet G, Tzoran I, **ITALY**: Abenante A, Barillari G, Basaglia M, Bilora F, Bissacco D, Bortoluzzi C, Brandolin B, Casana R, Ciammaichella MM, Dentali F, Di Micco P, Giorgi-Pierfranceschi M, Imbalzano E, Lambertenghi-Deliliers D, Marcon C, Martini R, Negro F, Poz A, Prandoni P, Scarinzi P, Simioni P, Siniscalchi C, Taflaj B, Tufano A, Visonà A, Vo Hong N, Zalunardo B, **LATVIA**: Skride A, Zicans M, **MOROCCO**: Tazi-Mezalek Z, **PORTUGAL**: Fonseca S, Gonçalves F, Marques R, Meireles J, Pinto S, **REPUBLIC OF NORTH MACEDONIA**: Bosevski M, Zdraveska M, **SWITZERLAND**: Bounameaux H, Mazzolai L, **UK**: Aujayeb A, **USA**: Bikdeli B, Caprini JA, Khalil A, Tafur J, Weinberg I, **VIETNAM**: Bui HM, Nguyen ST, Pham KQ, Tran GB.

**ACKNOWLEDGEMENTS**

We express our gratitude to **SANOFI** and **ROVI** for supporting this Registry with an unrestricted educational grant. We also thank the RIETE Registry Coordinating Center, S&H Medical Science Service, for their quality control data, logistic and administrative support.

**References**

1. Tritschler T, Kraaijpoel N, Girard P, Buller HR, Langlois N, Righini M, Schulman S, Segers A, Le Gal G, Subcommittee on P, et al. Definition of pulmonary embolism-related death and classification of the cause of death in venous thromboembolism studies: Communication from the SSC of the ISTH. *J Thromb Haemost*. 2020;18:1495-1500. doi: 10.1111/jth.14769

2. Schulman S, Kearon C, Subcommittee on Control of Anticoagulation of the S, Standardization Committee of the International Society on T, Haemostasis. Definition of major bleeding in clinical investigations of antihemostatic medicinal products in non-surgical patients. *J Thromb Haemost*. 2005;3:692-694. doi: 10.1111/j.1538-7836.2005.01204.x

3. Thygesen K, Alpert JS, Jaffe AS, Chaitman BR, Bax JJ, Morrow DA, White HD, Executive Group on behalf of the Joint European Society of Cardiology /American College of Cardiology /American Heart Association /World Heart Federation Task Force for the Universal Definition of Myocardial I. Fourth Universal Definition of Myocardial Infarction (2018). *Circulation*. 2018;138:e618-e651. doi: 10.1161/CIR.0000000000000617

4. Sacco RL, Kasner SE, Broderick JP, Caplan LR, Connors JJ, Culebras A, Elkind MS, George MG, Hamdan AD, Higashida RT, et al. An updated definition of stroke for the 21st century: a statement for healthcare professionals from the American Heart Association/American Stroke Association. *Stroke*. 2013;44:2064-2089. doi: 10.1161/STR.0b013e318296aeca

5. Konstantinides SV, Meyer G, Becattini C, Bueno H, Geersing GJ, Harjola VP, Huisman MV, Humbert M, Jennings CS, Jimenez D, et al. 2019 ESC Guidelines for the diagnosis and management of acute pulmonary embolism developed in collaboration with the European Respiratory Society (ERS). *Eur Heart J*. 2020;41:543-603. doi: 10.1093/eurheartj/ehz405

6. Jimenez D, Aujesky D, Moores L, Gomez V, Lobo JL, Uresandi F, Otero R, Monreal M, Muriel A, Yusen RD, et al. Simplification of the pulmonary embolism severity index for prognostication in patients with acute symptomatic pulmonary embolism. *Arch Intern Med*. 2010;170:1383-1389. doi: 10.1001/archinternmed.2010.199

7. Chesnaye NC, Stel VS, Tripepi G, Dekker FW, Fu EL, Zoccali C, Jager KJ. An introduction to inverse probability of treatment weighting in observational research. *Clin Kidney J*. 2022;15:14-20. doi: 10.1093/ckj/sfab158
